# Mesoscopic Mapping of Stimulus-Selective Response Plasticity in the Visual Pathways Modulated by the Cholinergic System

**DOI:** 10.3389/fncir.2020.00038

**Published:** 2020-07-03

**Authors:** Guillaume Laliberté, Rahmeh Othman, Elvire Vaucher

**Affiliations:** ^1^Laboratoire de Neurobiologie de la Cognition Visuelle, École d'Optométrie, Université de Montréal, Montréal, QC, Canada; ^2^Départment de Pharmacologie et Physiologie, Faculté de Médecine, Université de Montréal, Montréal, QC, Canada

**Keywords:** cholinergic potentiation, mesoscale calcium imaging, visual conditioning, acetylcholinesterase inhibitors, visual cortex

## Abstract

The cholinergic potentiation of visual conditioning enhances visual acuity and discrimination of the trained stimulus. To determine if this also induces long-term plastic changes on cortical maps and connectivity in the visual cortex and higher associative areas, mesoscopic calcium imaging was performed in head-fixed awake GCaMP6s adult mice before and after conditioning. The conditioned stimulus (0.03 cpd, 30°, 100% contrast, 1 Hz-drifting gratings) was presented 10 min daily for a week. Saline or Donepezil (DPZ, 0.3 mg/kg, s.c.), a cholinesterase inhibitor that potentiates cholinergic transmission, were injected prior to each conditioning session and compared to a sham-conditioned group. Cortical maps of resting state and evoked response to the monocular presentation of conditioned or non-conditioned stimulus (30°, 50 and 75% contrast; 90°, 50, 75, and 100% contrast) were established. Amplitude, duration, and latency of the peak response, as well as size of activation were measured in the primary visual cortex (V1), secondary visual areas (AL, A, AM, PM, LM, RL), retrosplenial cortex (RSC), and higher cortical areas. Visual stimulation increased calcium signaling in all primary and secondary visual areas, the RSC, but no other cortices. There were no significant effects of sham-conditioning or conditioning alone, but DPZ treatment during conditioning significantly decreased the integrated neuronal activity of superficial layers evoked by the conditioned stimulus in V1, AL, PM, and LM. The activity of downstream cortical areas was not changed. The size of the activated area was decreased in V1 and PM, and the signal-to-noise ratio was decreased in AL and PM. Interestingly, signal correlation was seen only between V1, the ventral visual pathway, and the RSC, and was decreased by DPZ administration. The resting state activity was slightly correlated and rarely affected by treatments, except between binocular and monocular V1 in both hemispheres. In conclusion, cholinergic potentiation of visual conditioning induced change in visual processing in the superficial cortical layers. This effect might be a key mechanism in the establishment of the fine cortical tuning in response to the conditioned visual stimulus.

## Introduction

**Figure 1:**
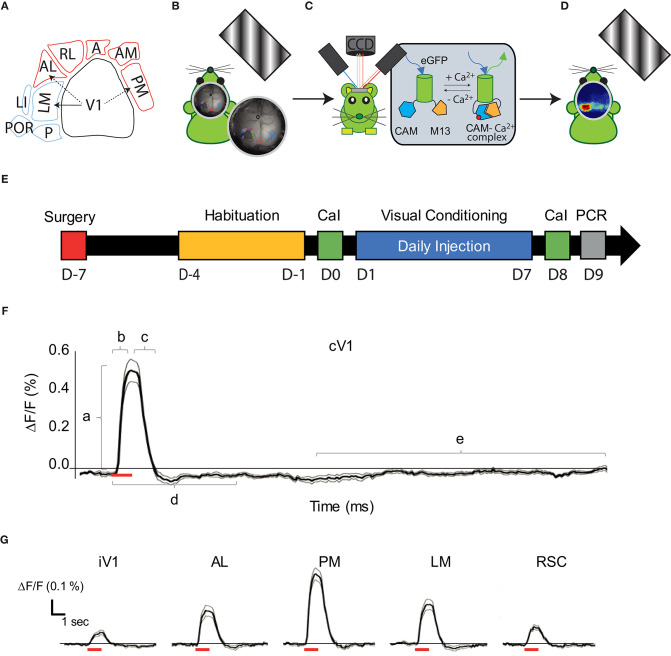
Visual pathways and methodology. **(A)** Schematic representation of the visual cortical areas adapted from (Zhuang et al., 2017) and their belonging to the dorsal (purple) or ventral (blue) stream. The density of V1's projections to LM, AL, and PM are greater (arrows) in these areas compared to adjacent areas, suggesting that LM and AL are the gateway for each visual stream. **(B)** Schematic representation of the experimental set-up: a monitor was placed on the right side (120°) of the head-fixed mouse to monocularly stimulate the right visual field by drifting oriented gratings. **(C)** A CCD camera was placed dorsal to the mouse skull to acquire light absorbance and fluorescent signal fluctuations in the cortex through an optic chamber. During the acquisition, the brain of the mouse was sequentially illuminated by three LED lights (see text for details). **(D)** Representation of cortical map in response to the monocular visual stimulation. **(E)** Timeline for the mice treatment and experimentation (mCaI, mesoscale Calcium Imaging; V.C., Visual Conditioning; RT-qPCR, retro-transcription quantitative polymerase chain reaction). **(F)** Trace of the mean CaS in function of time (*n* = 18 mice; 15 repetitions each, mean in dark, ± SEM in gray) in response to the 30 H visual stimulation (represented by a red bar) in cV1. Representation of the parameters examined is shown: *Amplitude* (a), *Latency* (b), *Persistence* (c), *Activation correlation* window (d) and the baseline (e). **(G)** Traces of the CaS mean response to the visual stimulation (red bar) in function of time (*n* = 18 mice; 15 repetitions each, mean in dark, ± SEM in gray), during the time window used for *activation correlation* calculation in iV1, AL, PM, LM, and RSC. cV1, contralateral primary visual cortex; iV1, ipsilateral primary visual cortex; PM, posterior-median cortex; LM, latero-median cortex; A, anterior cortex; AL, anterio-lateral cortex; AM, anterio-median cortex; RL: rostro-lateral cortex; RSC, retrosplenial cortex.

Vision is a primary sense that drives one's assessment of the external world and guides behavioral responses. Visual perception results from an interplay between various cortical areas. These areas are hierarchically organized, starting in the primary visual cortex (V1) (Glickfeld and Olsen, 2017). In mice, 12 associative visual areas, sharing close anatomical, and functional relationships with V1 (Wang and Burkhalter, 2007), process the information of complex visual stimuli. This processing starts with very selective responses of visual neurons for specific parameters of stimuli, such as orientation, spatial and temporal frequencies, and direction, which are associated with the visual hierarchy (Andermann et al., 2011). The functional selectivity of neurons and cortical areas defines visual pathways that follow a dorsal and a ventral stream in mice, as observed in greater mammals (Mishkin et al., 1982; Glickfeld et al., 2013a). The examination of circuitry between visual areas has revealed that the murine dorsal pathway, which sustains spatial perception, is composed of the latero-medial area (LM), laterointermediate area (LI), posterior area (P), and postrhinal area (Por) (Figure 1). The ventral pathway, which allows for the recognition of stimulus attributes, consists of the anterolateral area (AL), anterior area (A), anteromedial area (AM), rostrolateral area (RL), and posteriomedial area (PM) (Huberman and Niell, 2011; Wang et al., 2012). The dense projections from V1 to the LM and AL areas suggest that these areas could represent the entries of the dorsal and ventral visual pathways in mice, respectively.

The extraction of important visual information from the external environment requires neurons to respond with a differential strength and, consequently, involve specific visual microcircuits. A specific stimulus might require a high level of processing, thanks to neuronal gain modulation (Soma et al., 2012, 2013a) and neuronal plasticity, which would result in the persistent change of the neuronal response to this stimulus, as well as structural changes. Neuronal plasticity is defined as the principle of learning and the permanent improvement of perception. It is highly expressed in the developing brain, but it is rather latent after brain maturation when plasticity brakes such as Lynx1 (Morishita et al., 2010) are upregulated, or during the perineuronal net development (Hensch, 2005). Plasticity has to be reactivated in adults, specifically by manipulating the excitatory-inhibitory cortical balance via neuromodulation or by eliciting the long-term potentiation of the synapse strength. Also, neuronal plasticity could be reactivated via expression of plasticity factors that enhance plasticity, e.g., Lypd6 (Darvas et al., 2009; Sadahiro et al., 2016), or that structurize neuronal connectivity, such as the tissue plasminogen protein tPa (Mataga et al., 2002), and the synaptic proteins GAP43 (Han et al., 2013) or PSD95 (Kim and Sheng, 2004).

Stimulus-specific response plasticity is induced by conditioning in which a repetition of the stimulus enables the consolidation of neuronal reactivity. In the visual pathway, stimulus-specific response potentiation in V1 has been shown to involve gamma oscillations, the GABAergic microcircuits, and long-term potentiation of the response according to an Hebbian pattern (Cooke and Bear, 2010; Chen N. et al., 2012). It has also been shown to be enhanced by the cholinergic system (Kilgard and Merzenich, 1998; Rokem and Silver, 2013; Chen et al., 2015; Galuske et al., 2019; Vaucher et al., 2019), which strongly interacts with both the cortical GABAergic and glutamatergic microcircuits, inducing long-term potentiation-like mechanisms and refining circuitry efficiency. For these reasons, the cholinergic system has been proposed to be a key player in experience-induced plasticity. Acetylcholine (ACh) modulates the inhibitory GABAergic response through cholinergic nicotinic and muscarinic receptors (Mcclure-Begley et al., 2009; Disney et al., 2012; Demars and Morishita, 2014; Groleau et al., 2015). Additionally, ACh has multiple effects on the visual response, including effects on the latency (Turchi and Sarter, 1997), spread (Kimura et al., 1999; Voss et al., 2016), and signal gain (Minces et al., 2017) of the cortical response. From a behavioral point of view, it has been demonstrated that this neuromodulator enhances visual acuity (Kang et al., 2014) and recognition (Chubykin et al., 2013; Gavornik and Bear, 2014), as well as contrast detectability (Bhattacharyya et al., 2013; Soma et al., 2013c). These changes were measured in layer 4 of V1, or MT (Chen X. et al., 2012) in rodents and primates, and in associative areas. Notably, donepezil (DPZ, a cholinesterase inhibitor that potentiates cholinergic transmission) administration was found to reduce functional connectivity between cortical areas of the visual hierarchy in order to favor automated processing (Ricciardi et al., 2013). The cholinergic system controls cortical processing in defined cortical areas, though it can also coordinate cortical function as the cholinergic input comes from the basal forebrain. This cholinergic system sends wide but organized projections to the cortical mantle (Gaykema et al., 1990; Coppola et al., 2016; Huppé-Gourgues et al., 2018).

In the present study, the regional distribution of the effects of visual conditioning and DPZ was investigated in awake head-fixed Thy1-GCaMP6s mice. The goal of the study was to evaluate whether the cholinergic system would change the correlation of neural activity between areas to enhance efficiency and automation of the processing of the trained stimulus. The cholinergic system was potentiated through systemic administration of 0.3 mg/kg DPZ (Bontempi et al., 2003; Geerts et al., 2005; Bretin et al., 2018). A monocular conditioning to an oblique pattern was performed daily for a week. We used mesoscale calcium imaging (mCaI), which assesses the calcium influx from the excitatory (expressing Thy-1) neurons bodies and neurites of the superficial cortical layers (Chen et al., 2013; Dana et al., 2014), and allows for the establishment of whole-brain cortical maps. The focus was placed on the most reactive cortical areas, particularly V1, in both hemispheres, contralateral and ipsilateral to the stimulation (cV1 and iV1), as well as five areas of the ventral pathway (AL, A, AM, RL, and PM) that show great tuning for oriented gratings (Smith et al., 2017), and one area of the dorsal pathway (LM). The activity of the retrosplenial cortex (RSC) was assessed because of its role in contextual learning and memory (Makino and Komiyama, 2015; Leaderbrand et al., 2016). High-level areas were also analyzed but not reported as the signal was not significantly affected by the visual stimulation. Different parameters of the fluorescent calcium signal (CaS, representing ΔF/F, %) were measured: the *amplitude*, the *size* of the activated area, the signal-to-noise ratio (*SNR*), the *latency*, and the *persistence* of the maximal signal, which is indicative of the strength, rapidity and efficiency of the neural processing. The resting state functional connectivity was calculated before and after visual conditioning to assess the reorganization of circuitry efficiency. Finally, to investigate through which plasticity mechanisms the cholinergic enhancement and conditioning affect the visual cortex, the expression of Lypd6, Lynx1, tPa, GAP43, and PSD95 were examined by RT-qPCR after treatments.

## Materials and Methods

### Mice

All procedures were approved by the Animal Care Committee of the University of Montreal (CDEA, protocol 19-024) and conformed to the guidelines of the Canadian Council on Animal Care. Transgenic heterozygous GCaMP6s mice (*n* = 18, 9 males and 9 females equally distributed in three groups) were produced in our colony by breeding C57BL/6J-Tg(Thy1-GCaMP6s)GP4.3Dkim/J (IMSR Cat# JAX:024275, RRID:IMSR_JAX:024275) males with C57BL/6J wild type (IMSR Cat# JAX:000664, RRID:IMSR_JAX:000664) females, in agreement with the university's reproduction protocol (CDEA, 19-025). The GCaMP6s expression was determined by genotyping each animal with PCR amplification, in accordance with the Jackson Laboratory (RRID:SCR_004633) procedures for this strain. Mice were kept in a 12 h-light cycle room with *ad libitum* access to food and water. To prevent any potential bias caused by the circadian cycle, daily experiments (habituation, mCaI acquisition, drugs administration, and visual conditioning) were performed within the same daily time period (between 8 and 12 AM) for each mouse. The testing order was randomly determined on the first day using one mouse from each experimental group [non-conditioned group [Sham], control conditioned [CS], or 0.3 mg/kg DPZ conditioned [CS/DPZ] group; six series of experiments were performed]. Three (one in each group) out of the 18 initial mice were removed after complications following the surgery or during the treatment, thus there were *n* = 5 per group.

### Surgical Procedures

For chronic implantation of the imaging chamber, animals were anesthetized with isoflurane (induction at 5%, maintain in 1.5%; in medical *O*_2_) and placed in a stereotaxic frame. Body temperature was maintained at 37°C using a heating pad and monitored with a rectal thermometer throughout the procedure. The scalp was shaved, decontaminated with ethanol (70% v/v) and iodine (16% v/v), and locally anesthetized with subcutaneous injection of lidocaine (32 mg/kg). The skin covering the skull was removed and replaced with transparent dental cement (C&B MetaBond, Parkell, Edgewood, NY, USA), a cover glass (Carolina, Burlington, NC, USA), and an 11 mm diameter titanium head fixation chamber. At the end of the procedure, mice were injected subcutaneously with a non-steroidal anti-inflammatory drug, carprofen (0.5 mg/kg), in saline (injection volume equivalent to a ratio of 0.1 mL/10 g of the mouse's weight) solution and allowed to recover for 30 min in a red-light warmed cage. They were then placed individually in a clean cage. A second and third subcutaneous injection of carprofen (0.5 mg/kg) were performed at 24 and 48 h after the surgery. The animal was allowed to recover 5 days after the surgery before beginning any head-fixed procedure.

### Mesoscale Calcium Imaging Recordings

The fluorescent CaS recording was performed on awake head-fixed mice at day 0 (D0, before the conditioning) and day 8 (D8, 1 day after conditioning) (Figure 1). No recording of the CaS in response to a visual stimulation was performed during conditioning (7 consecutive days). For the resting state cortical activity, the CaS was acquired at D0 and D8 during a period of 10 min prior to visual stimulation.

During mCaI acquisition, the mice stand inside a fenestrated PVC tube on a height-adjustable stage placed within a dark cabinet. A computer monitor (60 Hz refresh time; 250 cd/m^2^, main luminance) was positioned at 21 cm from the mouse's side (120°) to stimulate its entire monocular visual field (Figure 1B). To minimize stress, mice were progressively habituated to the head-fixation apparatus over 5 days: 5, 10, 20, and 40 min head fixation periods without brain illumination for the first 4 days, respectively, and then 40 min with brain illumination and gray screen presentation for the last day of habituation. This habituation abolished signs of stress in the cage and on the stage (mice showed adequate grooming, diminution of vocalization, diminution of movements during the head fixation, and no weight loss). The mice were placed in a dark room for 30 min prior any CaI recording including 5 min head-fixation at rest. The CaI recording was synchronized to the visual stimulation with the Datapixx3 device (Vpixx Technologies Inc., St Bruno, QC, Canada).

The CaS was recorded through a CCD camera (NIKKOR 50 mm f/1.2, Nikon, Minato, Tokyo, Japan) positioned vertically above the skull (Figure 1C). A dark opaque screen was placed between the monitor displaying the visual stimulation and the imaging chamber/camera to ensure there was no light contamination. Sequential 472, 590, and 623 nm brain illumination was produced by three LEDs contained in two adjustable illumination arms (Figure 1C) directly on the skull of the mouse. Calcium indicators were excited at 472 nm (Blue LED, Cree XLamp XP-E2 LEDs, Cree, Durhamm, NC, USA) and intrinsic signals (absorbance of oxy- and deoxy- hemoglobin) were extracted from modifications in the absorption of the 590 and 623 nm wavelengths (Amber LED, LZ4-00MA00 and Red LED, LZ4-00MA00, respectively, OSRAM, Markham, Ontario, Canada). The reflectance of intrinsic signals and the fluorescence emission were collected at a frame rate of 30 Hz (10 Hz by wavelength) with a full resolution of 512 × 512 pixels (21.5 μm/pixel). Illumination was adjusted to avoid under or over saturation of any wavelengths. The exposure time of the camera was set to 18 ms. Filters (long pass filter at 496 nm adjusted to the objective) were used to minimize any contamination from other wavelengths.

### Image Processing and Analysis of Calcium Signals

**Figure 2:**
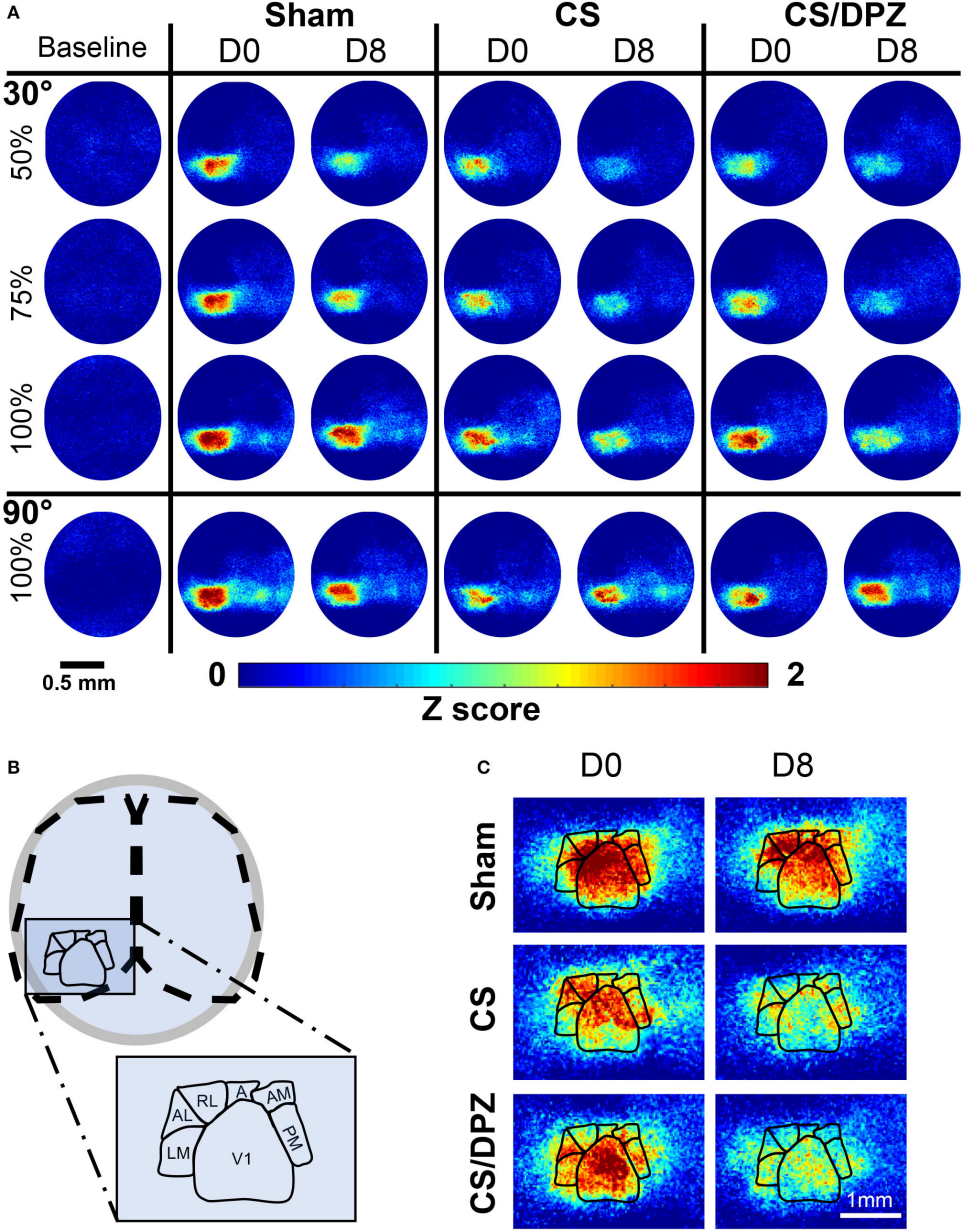
Cortical activation maps of adult Thy1-GCaMP6s mice. **(A)** Color-coded maps show the effect of a sham-conditioning (Sham) visual conditioning coupled with saline (CS) or cholinergic potentiation (CS/DPZ) on the cortical response in superficial layers. Response to a conditioned oriented visual stimulation in different contrast (30 L, 50%, 30 M, 75%, and 30 H, 100%, top panel) and a non-conditioned orientation (90 H, 100%, bottom panel) are represented at day 0 (D0) and day8 (D8) after the conditioning. The cortical response to the conditioned stimulus and its lower contrast- equivalent is reduced for both injection groups (Data represented in *z*-score median, *n* = 5/experimental groups). **(B)** Schematic representation of the ROI mask used to extract CaS from the different visual cortices. **(C)** Magnification of the visual areas (for the 30 H stimulation). The reduction of cortical response has a general occurrence across the visual areas, while the ventral stream (AL, RL, A, AM, and PM) express more initial (D0) activation than the dorsal path (LM) for the visual stimulation.

All data were imported and analyzed with Labeo Technologies, Inc. and MATLAB codes (MathWorks, Natick, MA, USA, RRID:SCR_001622). Prior to data analysis, recorded images of the whole cortex were corrected for the camera's electronic noise. In addition, each pixel's intensity time course was filtered with a low pass filter to remove high frequency artifacts related to respiration and heart rate. Pixels were fused 1:2, so CaI analysis was performed over a 256 × 256 pixel window (43 μm/pixels). Tissue absorbance due to the hemodynamic response (assessed by the at 590 and 623 nm illumination) was subtracted from the fluorescence signal using a modified Beer-Lambert equation (Guevara et al., 2013). The corrected CaS (ΔF/F, %) of each pixel was normalized by subtracting the current CaS with the CaS baseline over the CaS baseline × 100. The CaS of each pixel (CaS_p_) was then spatially normalized using the Z-score to create cortical activity maps (Gias et al., 2005) (Figures 1D, 2).

$$\begin{array}{l} Z-score=\;\frac{CaS_{p}-mean\left(CaS_{p}\right)}{SD\left(CaS_{p}\right)} \end{array}$$

The size and position of the regions of interest (ROIs) were adapted from the Allen Brain Institute (Zhuang et al., 2017) and other anatomical and functional studies imaging the mouse visual cortex (Andermann et al., 2011; Marshel et al., 2011; Wang et al., 2012; Glickfeld et al., 2013a; Groleau et al., 2014; Wekselblatt et al., 2016). A ROI template was generated, manually centered, and fitted to Bregma and Lambda (Figures 1B, 2B). The size and position of the ROIs were automatically corrected according to the Bregma and Lamda distance for each mouse at D0 to avoid misidentification and cross-contamination between secondary visual areas. The CaS of each pixel contained in the ROIs was averaged in response to each visual stimulation (15 times). The resultant ROIs' CaS was then averaged across animals (*n* = 18). The CaS response in a particular ROI was considered an outlier and removed from the analysis when the CaS simultaneously measured in control areas (primary motor and somatosensory cortex) varied from the mean CaS calculated across the whole cortex (calculated with a 95% confidence interval).

Various stimulation parameters were calculated (Figure 1F):

The *Amplitude* was calculated by subtracting the baseline recorded during gray screen presentation from the maximal fluorescent signal during the stimulus presentation.

$$\begin{array}{l} Amplitude=CaS_{Max}-CaS_{Baseline} \end{array}$$

The *Size* was defined as the surface (number of pixels) of each ROI activated by the visual stimulation. We considered that the ROI was activated when the z-score of the pixel's CaSmax was >1.282. The number of activated pixels was normalized by the surface of the ROI to minimize the impact of the ROI dimensions.

$$\begin{array}{l} Size=\frac{Pixels_{\;Z-score\;>1.282}}{Pixels_{\;Total\;in\;ROI}} \end{array}$$

The *Latency* represented the time interval from the beginning of the visual presentation to CaS_Max_.

The *Persistence* was the time interval between the end of the stimulation presentation and the end of the CaS, i.e., when the fluorescent signal corresponded to <2 SD of the baseline.

$$\begin{array}{l} Persistence\;\left(ms\right)\to \;CaS\;\leq \;\left(CaS_{Baseline}+2\sigma_{Baseline}\right) \end{array}$$

The *signal-noise ratio* (SNR) of local activation was calculated by measuring the ratio between the maximal response for the stimulation and the standard deviation of the signal baseline.

$$\begin{array}{l} SNR=\frac{\left(CaS_{Max}\right)}{\sigma_{Baseline}} \end{array}$$

The *activation correlation* between the nine ROIs was determined using the MATLAB function *corrcoef* over a 40 ms window starting at the beginning of the stimulus presentation and represented by a matrix.

The *resting state correlation* was determined by measuring the cross-correlation coefficient r values between the temporal profiles of each of the 11 seed pixels (LM, V1b, V1m, AL, PM, RL, A, AM, RSC, AC, and M1 where AC and M1 are the anterior cingulate cortex and primary motor cortex, respectively) in each hemisphere (and all others over 10 min of resting state acquisition). Their locations were defined according to the Allen Institute Atlas (Allen Reference Atlas—Mouse Brain, RRID:SCR_013286) and were corrected by the distance between the manually selected Bregma and Lambda as previously described.

### Visual Stimulation

The visual stimulation provided during CaI acquisition consisted of a series of drifting gratings (spatial frequency: 0.03 cpd, temporal frequency: 1 Hz, orientation: 30 or 90°, contrast: 50, 75, or 100 %) produced by a Vpixx software (Vpixx Technologies Inc.) and displayed on an LCD screen (23″ ACER LCD monitor S230HL, Refresh Rate 60 Hz, Brightness 250 cd/m^2^) positioned in the right monocular field at 21 cm from the mice (Figure 1B). Each stimulus was randomly presented 15 times during 1 s with 25 s of inter-stimulation intervals (gray screen). The CaS parameters were calculated for each distinct visual stimulation condition.

### Visual Conditioning

The visual conditioning of awake head-fixed mice consisted of a unilateral left exposure to a specific stimulus every day over 7 consecutive days (Figure 1E). A unilateral presentation of a gray screen was used for the non-conditioned group, while a drifting grating (S.F.: 0.03 cpd, T.F.: 1 Hz, Ori.: 30°, Con.: 100%) was presented to both conditioned groups during 50 s for 12 presentations with 10 s intervals of presentation (for a total stimulation time of 10 min).

### Drug Administration

Mice were injected subcutaneously behind the neck with 0.1 mL/10 g (mouse weight) of sterile saline (Sham and CS, *n* = 5 per group) or with 0.3 mg/kg DPZ (Bontempi et al., 2003; Bretin et al., 2018) diluted in 0.1 mL/10 g (mouse weight) of sterile saline (CS/DPZ; *n* = 5) 15 min prior to the visual conditioning session in order to reach the maximal cortical effect of the drug (Geerts et al., 2005).

### RNA Extraction

Immediately after the last CaI session, mice were deeply anesthetized with pentobarbital and sacrificed by decapitation. The brain was collected on a cold plate and placed in RNAlater stabilization reagent (QIAGEN, Valencia, CA, USA) for 24–48 h. The contralateral primary visual cortex (1 mm^3^ centered on Bregma: −4 mm, Interaural: 2.5 mm) was dissected on ice within 60 s with RNAzap-treated instruments and stored at −80°C. Total RNA was extracted from the contralateral V1 using Qiazol reagent and the RNeasy®Lipid Tissue Mini Kit (QIAGEN, Valencia, CA, USA) according to the manufacturer's protocol. RNA concentration was determined using Nanodrop (ThermoFisher Scientific, Waltham, MA, USA), measuring 260/280 nm and 260/230 nm absorbance ratios. Real-time qPCR of 80 ng extracted RNA and specific primers (Table 1) was made using Quantifast® SYBR® Green RT-qPCR (QIAGEN, Valencia, CA, USA) using the manufacturer's protocol. Both targeted and referenced genes were amplified in duplicate on the same run. The relative quantification of each gene was determined using the MxProTM Q-PCR software version 3.00 (Stratagene, La Jolla, CA, USA), where the average of each duplicate mRNA levels was normalized by the 2^−ΔΔ*Ct*^ methods (Livak and Schmittgen, 2001) using housekeeping genes 18S and the non-conditioned group (naïve animal) as a control.

**Table 1:** Primers list.

		**Sequence**
GCaMP6s	Forward	5′-ACA AGC AGA AGA ACG GCA TC-3′
	Reverse	5′-TGG TAG TGG TAG GCG AGC TG-3′
18S	Forward	5′-GTA ACC CGT TGA ACC CCA TT-3′
	Reverse	5′-CCA TCC AAT CGG TAG TAG CG-3′
Lynx1	Forward	5′-CCA CCT ACT GTA TGA CCA CAC G-3′
	Reverse	5′-CAA CAG CAG GTG GCA GAT GCA T-3′
Lypd6	Forward	5′-CAC TCC GTA TCC TGG TGG GTT T-3′
	Reverse	5′-GAC TTC CAT CGT GTG CTG AGT G-3′
tPa	Forward	5′-TGG TGC TGT TGG TAA GTT GT-3′
	Reverse	5′-TGC CTG ACC AGG GAA TAC AT-3′
PSD95	Forward	5′-TCA ACA CGG ACA CCC TAG AA-3′
	Reverse	5′-TGA GTT ACC CCT TTC CAA TG-3′
GAP43	Forward	5′-TGG AAC AAG ATG GTG TCA AG-3′
	Reverse	5′-CCT TTG AGC TTT TTC CTT GT-3′
M2	Forward	5′-AAG TCA ACC GCC ACC TTC AGA C-3′
	Reverse	5′ GTA GCC AAT CAC AGT GTA GAG GG-3′

### Statistical Analysis

The data were analyzed using GraphPad Prism software version 8 (GraphPad Software, San Diego, CA, USA). For the first experiment assessing sensitivity of the CaS to contrast and orientation (*n* = 18), outliers were detected and removed using the ROUT method (Q = 1%) (Motulsky and Brown, 2006). To determine whether there was a significant modification in the area responses (*Amplitude, Size, Latency, Persistence*, and *SNR)* for the stimuli contrast (L, M, H), a Kruskal-Wallis test was performed individually for both orientations (30° and 90°) in each area (cV1, iV1, PM, LM, RL, A, AM, AL, and RSC). To evaluate the difference between both orientation (30 and 90 H) responses, we used a one-tail Wilcoxon test as we expected a better response at 90°.

For the conditioning experiment, to investigate the treatments (i.e., Sham, CS and CS/DPZ, *n* = 5 per group) and effects on the cortical response, we used the Wilcoxon matched pairs signed rank test on pre and post-conditioning responses for both orientations (30 vs. 90 H). Then, Kruskal-Wallis and uncorrected Dunn's tests were used to compare experimental groups in terms of post-pre responses for each stimulation pattern.

The *activation-* and *resting state correlations* were normalized using the Fisher Z-Transformation, then compared pre vs. post effects using *t*-tests (*n* = 5). A *t*-test was used to compare the post-conditioning of both conditioned groups. To enhance the clarity of the connectivity matrix, the heatmap was reorganized by putting high r values closer to the diagonal line using the *reorderMAT* function (Brain Connectivity Toolbox, RRID:SCR_004841) on the pre-conditioning (30 H) activation correlation (*n* = 18) and resting state (*n* = 18) matrices.

The normal distribution of RT-qPCR data was confirmed with the Kolmogorov-Smirnov test and compared using a two-way ANOVA and the Bonferroni's multiple comparisons test. Results were illustrated using bars graph representing mean ± S.E.M., with a Pearson-coefficient correlation heatmap for clarity.

The statistical analysis was not corrected for multiple comparisons since this correction could lead to robust under-evaluation of changes for a large number of comparisons (Rothman, 1990), as required for the statistical analysis of multiple cortical regions as seen here. All of the data and statistical results were presented instead. The data sets generated and/or analyzed during the current study are available from the corresponding author upon reasonable request.

## Results

### Selectivity of the Cortical Response Assessed by Mesoscale Calcium Imaging

The sensitivity of the CaS to various contrasts or orientations was first evaluated in naïve animals (*n* = 18), in nine selected cortical areas involved at different levels of visual processing (cV1, iV1, PM, LM, RL, A, AM, AL, and RSC). These areas were selected because of their responsiveness to the stimulation (0.03 cpd, 1 Hz sinusoidal grating). The responsiveness of other cortical areas was negligible and not reported. Note that the calcium signal in this Thy1-GCAMP6s line of mice mainly arises from the excitatory neurons and neurites of the superficial layers, although the GCaMP6s marker is expressed by 80% of pyramidal neurons in cortical layers 2/3 and 5 (Dana et al., 2014). The CaS is negligible in GABAergic cells. Due to the density of the cortical tissue, the fluorescent signal from the superficial layers will have a stronger influence on the acquired signal compared to the signal from deeper layers, which will be more diffuse (Ma et al., 2016). Different parameters were assessed to detect any change in neuronal encoding, i.e., the amplitude of the signal response (*Amplitude*) (Hendel et al., 2008), the proportion of the activated area (*Size*) (Kimura et al., 1999), the time before the maximal response (*Latency*) (Mentis et al., 2001), the persistence of the calcium response (*Persistence*) after the stimulus presentation, and the Signal-Noise ratio (*SNR*) (Rieke et al., 1999).

**Figure 3:**
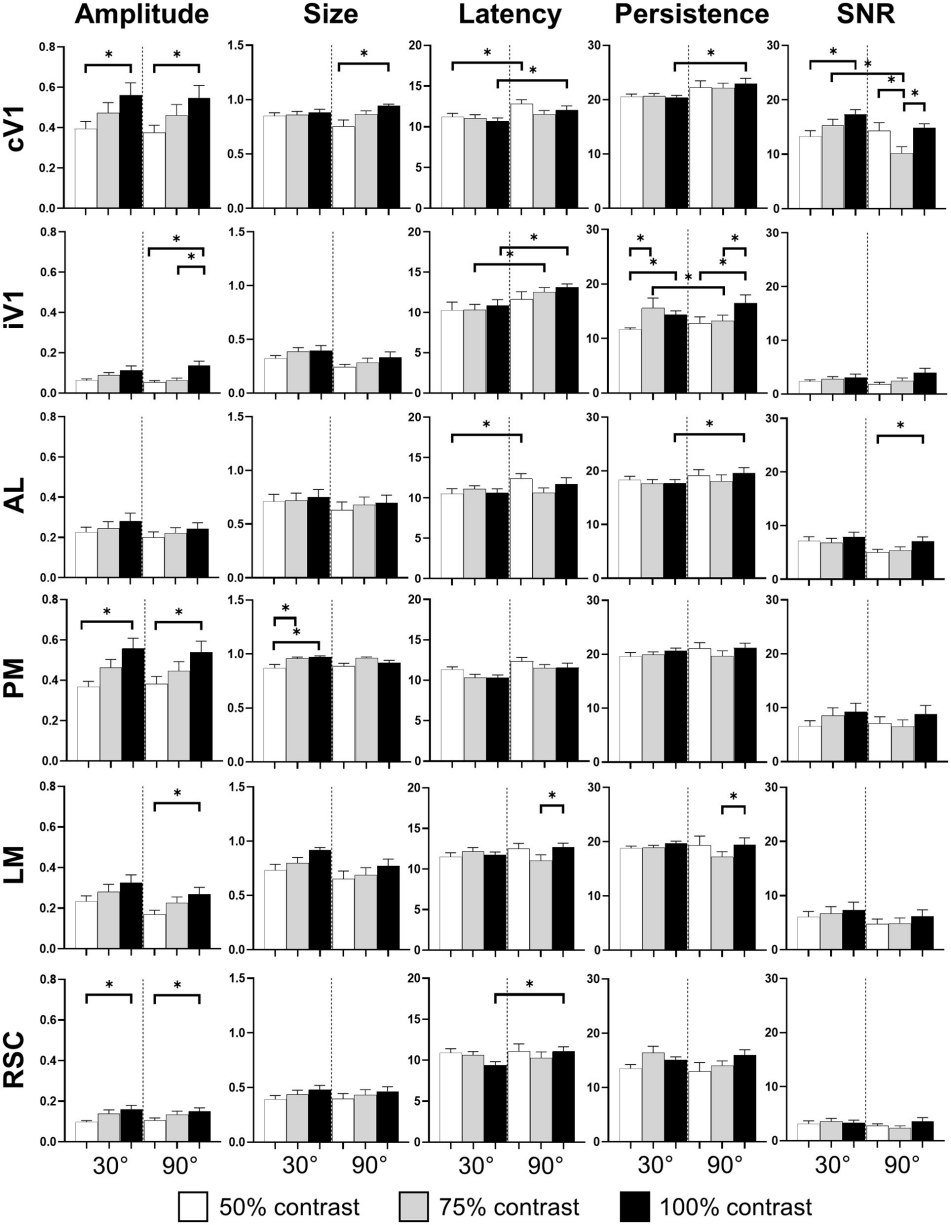
Cortical response in function of orientation and contrast of the stimulus. The contrast of the stimulation influences the amplitude response (*Amplitude*) in almost every area. The proportion of area activated (*Size*) seems to be influenced by the contrast only in PM. The response latency (*Latency*) is influenced by the grating orientation in V1 and RSC while the response duration (*Persistence*) is only influence in V1 by this stimulus parameter. Finally, contrast and orientation of the grating influence the signal-noise ratio (*SNR*) only in V1 (*n* = 18, Kruskal-Wallis and multiple *t*-test, *p < 0.05).

The pattern visual stimulation elicited a CaS increase in the majority of the observed areas, which was not significantly different between the stimulation conditions (orientation or contrast) in AL, AM, LM, RL, and RSC, but significantly affected by the stimulation conditions in PM and V1 according to the Kruskal-Wallis analysis. An increase in neuronal activity (*amplitude*) upon visual stimulation was detected in all examined regions (Table 2, Figures 2A, 3). Visually induced CaS was sensitive to contrast, particularly in cV1, iV1, PM, LM, and RSC (Figure 3). The *amplitude* of the CaS was identical for the two orientations of the grating (30° or 90°). The other parameters studied were rarely affected by the contrast or orientation changes, though some isolated significant changes were detected: the s*ize* was significantly increased in PM for the 30° orientation, and in cV1 for the 90° orientation with higher contrast of the stimulation (Table 2, Figure 3), the *latency* of the peak response was dependent on the orientation in certain cortical areas (V1, AL, and RSC), and the 90° orientation induced a higher latency of the CaS_Max_. Our results showed that the *Persistence* was also significantly higher for the 90° orientation in V1 and AL (Table 2, Figure 3). *SNR* was affected by the contrast for both orientations only in cV1 (Table 2, Figure 3).

**Table 2:** Response parameters in function of the stimulation contrast and orientation.

**Area**	**Stim**.	**Amplitude (ΔF/F, %)**	**Size (Prop.)**	**Latency (ms)**	**Persistence (ms)**	**SNR**
cV1	30 L	**0.39 ± 0.04**	**0.85 ± 0.03**	11.22 ± 0.44	20.59 ± 0.45	**13.26 ± 1.08**
	30 M	0.47 ± 0.05	0.86 ± 0.03	11.06 ± 0.44	20.69 ± 0.44	15.26 ± 1.18
	30 H	0.56 ± 0.06	0.88 ± 0.03	10.72 ± 0.38	20.41 ± 0.38	17.33 ± 0.88
	90 L	**0.38 ± 0.04**	0.75 ± 0.06	12.83 ± 0.50	22.25 ± 1.20	14.32 ± 1.50
	90 M	0.46 ± 0.06	0.86 ± 0.03	11.50 ± 0.51	22.13 ± 0.90	10.17 ± 1.25
	90 H	0.55 ± 0.06	0.95 ± 0.01	12.06 ± 0.51	22.94 ± 0.99	14.81 ± 0.79
PM	30 L	**0.37 ± 0.03**	**0.87 ± 0.03**	11.33 ± 0.33	19.59 ± 0.76	6.53 ± 1.05
	30 M	0.46 ± 0.04	0.96 ± 0.01	10.33 ± 0.40	19.94 ± 0.51	8.51 ± 1.49
	30 H	0.56 ± 0.05	0.97 ± 0.01	10.33 ± 0.32	20.63 ± 0.54	9.22 ± 1.62
	90 L	**0.38 ± 0.04**	0.89 ± 0.03	12.33 ± 0.50	21.07 ± 1.10	7.08 ± 1.25
	90 M	0.45 ± 0.04	0.96 ± 0.01	11.50 ± 0.47	19.63 ± 1.03	6.48 ± 1.28
	90 H	0.54 ± 0.05	0.92 ± 0.02	11.56 ± 0.57	21.18 ± 0.89	8.76 ± 1.67
LM	30 L	0.23 ± 0.03	0.73 ± 0.06	11.50 ± 0.51	18.80 ± 0.39	6.06 ± 1.02
	30 M	0.28 ± 0.04	0.80 ± 0.05	12.17 ± 0.47	18.88 ± 0.46	6.69 ± 1.26
	30 H	0.32 ± 0.04	0.92 ± 0.02	11.72 ± 0.37	19.71 ± 0.40	7.36 ± 1.46
	90 L	**0.17 ± 0.02**	0.65 ± 0.08	12.50 ± 0.65	19.35 ± 1.68	4.77 ± 0.92
	90 M	0.23 ± 0.03	0.69 ± 0.07	11.06 ± 0.70	17.19 ± 0.98	4.87 ± 1.01
	90 H	0.27 ± 0.03	0.72 ± 0.06	12.71 ± 0.49	19.41 ± 1.30	6.16 ± 1.23
A	30 L	0.18 ± 0.02	0.34 ± 0.08	10.06 ± 1.00	15.59 ± 0.91	3.60 ± 0.70
	30 M	0.20 ± 0.04	0.40 ± 0.08	8.50 ± 0.44	14.18 ± 1.49	3.90 ± 0.94
	30 H	0.20 ± 0.04	0.33 ± 0.07	9.83 ± 0.82	14.00 ± 1.21	3.10 ± 0.61
	90 L	0.17 ± 0.03	0.21 ± 0.03	11.39 ± 0.70	14.53 ± 1.76	2.57 ± 0.52
	90 M	0.18 ± 0.03	0.33 ± 0.07	11.72 ± 0.80	15.00 ± 1.61	2.54 ± 0.48
	90 H	0.22 ± 0.03	0.30 ± 0.07	11.17 ± 0.97	16.71 ± 1.23	3.36 ± 0.69
AL	30 L	0.27 ± 0.03	0.71 ± 0.07	10.56 ± 0.62	19.07 ± 0.38	5.52 ± 0.96
	30 M	0.31 ± 0.05	0.72 ± 0.07	10.94 ± 0.45	18.36 ± 0.52	5.45 ± 1.01
	30 H	0.36 ± 0.06	0.84 ± 0.05	10.50 ± 0.47	17.25 ± 0.82	5.59 ± 1.08
	90 L	0.24 ± 0.04	0.63 ± 0.08	12.33 ± 0.67	17.76 ± 1.57	3.99 ± 0.72
	90 M	0.28 ± 0.05	0.68 ± 0.07	11.17 ± 0.61	16.53 ± 1.56	4.84 ± 0.79
	90 H	0.33 ± 0.05	0.70 ± 0.07	11.94 ± 0.78	19.59 ± 1.12	5.74 ± 1.03
AM	30 L	0.20 ± 0.02	0.37 ± 0.07	10.44 ± 0.79	17.53 ± 0.94	4.02 ± 0.75
	30 M	0.23 ± 0.03	0.44 ± 0.06	9.78 ± 0.72	18.18 ± 1.21	4.69 ± 1.04
	30 H	0.25 ± 0.04	0.41 ± 0.06	10.61 ± 0.70	16.47 ± 1.33	4.18 ± 0.79
	90 L	0.21 ± 0.03	0.40 ± 0.07	12.00 ± 0.67	17.69 ± 1.78	3.94 ± 0.84
	90 M	0.22 ± 0.04	0.39 ± 0.07	12.28 ± 0.66	19.00 ± 1.54	2.93 ± 0.52
	90 H	0.26 ± 0.04	0.34 ± 0.06	12.00 ± 0.94	18.29 ± 1.42	4.45 ± 0.979
RL	30 L	0.21 ± 0.02	0.59 ± 0.07	9.72 ± 0.80	17.41 ± 0.62	4.38 ± 0.76
	30 M	0.20 ± 0.02	0.64 ± 0.08	11.67 ± 0.56	18.18 ± 0.69	4.86 ± 1.00
	30 H	0.25 ± 0.04	0.63 ± 0.05	11.67 ± 0.56	16.88 ± 0.69	4.53 ± 0.86
	90 L	**0.17 ± 0.02**	0.56 ± 0.06	11.61 ± 0.81	17.53 ± 1.54	3.52 ± 0.63
	90 M	0.23 ± 0.03	0.59 ± 0.07	10.28 ± 0.74	16.69 ± 1.12	3.08 ± 0.56
	90 H	0.27 ± 0.03	0.58 ± 0.07	11.61 ± 0.82	18.47 ± 1.01	4.74 ± 0.93
RSC	30 L	**0.10 ± 0.01**	0.39 ± 0.03	10.89 ± 0.52	13.53 ± 0.72	3.16 ± 0.52
	30 M	0.14 ± 0.02	0.44 ± 0.04	10.59 ± 0.46	16.41 ± 1.21	3.52 ± 0.62
	30 H	0.16 ± 0.02	0.48 ± 0.04	9.39 ± 0.43	15.06 ± 0.59	3.28 ± 0.52
	90 L	**0.10 ± 0.01**	0.39 ± 0.05	11.06 ± 0.93	12.94 ± 1.67	2.74 ± 0.40
	90 M	0.13 ± 0.02	0.43 ± 0.05	10.28 ± 0.74	14.07 ± 0.85	2.36 ± 0.40
	90 H	0.15 ± 0.02	0.46 ± 0.04	11.06 ± 0.59	15.94 ± 1.02	3.62 ± 0.67

*Values represent parameters (Amplitude, Size, Latency, Persistence and SNR) of response to the visual stimulation (30 L: 30°, 50%; 30 M: 30°, 75%; 30 H: 30°, 100%; 90 L: 90°, 50%; 90 M: 90°, 75%; 90 H: 90°, 100%) (means ± S.E.M.), 30 L or 90 L. Bold characters represent significant value. p ≤ 0.05 compared to low contrast counterparts; Underline characters represent significant values comparing 30H response to 90H. p ≤ 0.05 compared to 90 H*.

### Cortical Activation Mesoscale Maps After a Visual Conditioning Coupled With Saline or Cholinergic Potentiation

**Figure 4:**
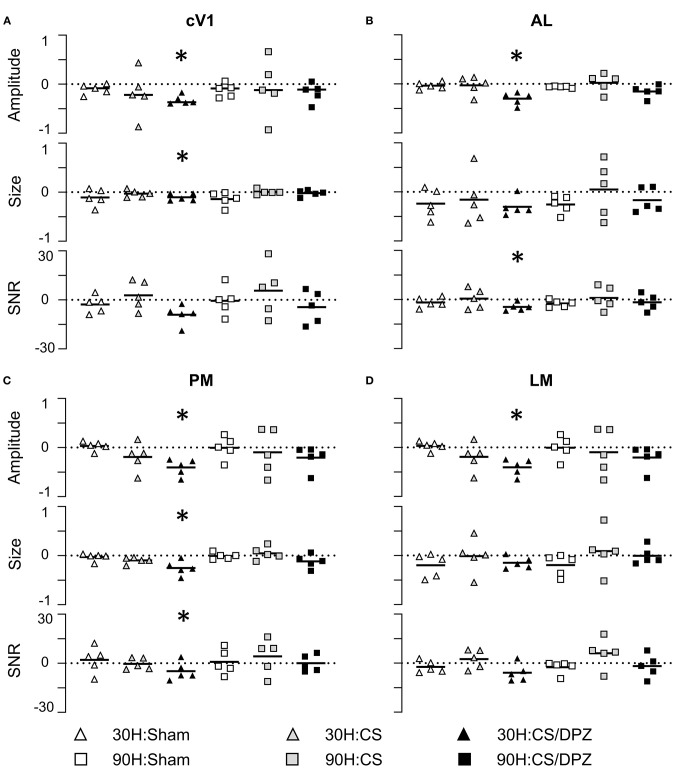
Conditioning and cholinergic potentiation effect on the cortical calcium response in cV1, AL, LM, and PM areas for the conditioned stimulus 30 H and non-conditioned stimulus 90 H After conditioning, the *Amplitude* showed a significant decrease for the conditioned stimuli in cV1 **(A)**, AL **(B)**, PM **(C)**, and LM **(D)** for the CS/DPZ group but not the other conditions. The *Size* showed a significant decrease for the conditioned stimuli (30 H) in cV1 **(A)** and PM **(C)** for the CS/DPZ group. The *SNR* showed a significant decrease for the conditioned orientation in cV1 **(A)**, AL **(B)**, and in PM **(C)** (30 H) for the CS/DPZ group (*n* = 5 for each group; One tail Wilcoxon test, *p < 0.05, comparing D8 to D0, one tail Wilcoxon test).

The effect of the passive 1-week monocular visual conditioning associated or not with cholinergic potentiation on the cortical calcium response features (*Amplitude, Size, Latency*, and *SNR*) was then examined, as well as sham-conditioning (gray screen instead of gratings) (Figure 4, Tables 3–6). The normalized activation maps (via spatial Z-score transformation, see methods) showed a clear and localized activation of contralateral visual cortices in response to each visual stimulation for all of the mice. The ipsilateral cortex activity was not altered, except occasionally and faintly in the bilateral part of V1. The post-conditioning CaS *Amplitude* values had a tendency to decrease compared to pre-values in all contralateral cortical areas for the CS/DPZ group, with the exception of stimulation contrast in cV1 (Figure 4A) and PM (Figure 4C) and for the conditioned stimulus in AL (Figure 4B) and LM (Figure 4D). The *Amplitude* for the conditioned stimulus in the CS and sham groups decreased only for the lowest stimulation contrast (30 and 90 L) after the conditioning, and there was no change in response to the non-conditioned stimulus (Table 3). The post-conditioning *Size* (Table 4) only decreased in cV1 (Figure 4A) and PM (Figure 4C). The other parameters were virtually unaffected by the conditioning, except for the *SNR* (**Table 6**) which was affected in the secondary visual area AL (Figure 4B) and PM (Figure 4C) in the CS/DPZ group. The changes observed for the lower contrast of 50%, even for Sham animals (Tables 3–6), were considered irrelevant as mice have poor visual acuity at this contrast, and that this low contrast pattern stimulus might be seen as a gray screen. Thus, the gray screen presented to the non-conditioned group might behave like a conditioned stimulus.

**Table 3:** Modification in the *Amplitude* (ΔPost-Pre cortical response) of the visual stimulation.

**Area**	**Stim**.	**Treatment**					
		**Sham**		**CS**		**CS/DPZ**	
cV1	30 L	**−0.188 ± 0.007**	**(−0.188)**	**−0.214 ± 0.059**	**(−0.162)**	**−0.106 ± 0.018**	**(−0.116)**
	30 M	**−0.193 ± 0.013**	**(−0.186)**	−0.190 ± 0.076	(−0.078)	**−0.198 ± 0.036**	**(−0.158)**
	30 H	−0.179 ± 0.024	(−0.205)	−0.188 ± 0.074	(−0.088)	**−0.216 ± 0.043**	**(−0.145)**
	90 H	−0.195 ± 0.021	(0.198)	−0.043 ± 0.084	(0.168)	−0.041 ± 0.038	(−0.098)
iV1	30 L	−0.012 ± 0.008	(−0.020)	−0.011 ± 0.020	(0.000)	0.055 ± 0.040	(0.008)
	30 M	−0.043 ± 0.014	(−0.042)	0.020 ± 0.012	(0.030)	−0.053 ± 0.012	(0.012)
	30 H	−0.023 ± 0.031	(0.010)	0.018 ± 0.015	(−0.006)	−0.023 ± 0.009	(−0.005)
	90 H	−0.058 ± 0.019	(−0.088)	−0.014 ± 0.033	(0.006)	−0.040 ± 0.020	(0.031)
AL	30 L	**−0.120 ± 0.018**	**(−0.123)**	−0.115 ± 0.045	(−0.072)	−0.074 ± 0.024	(−0.028)
	30 M	−0.099 ± 0.028	(−0.122)	−0.062 ± 0.060	(−0.003)	−0.143 ± 0.033	(−0.157)
	30 H	−0.057 ± 0.031	(−0.072)	−0.067 ± 0.059	(0.028)	**−0.204 ± 0.037**	**(−0.209)**
	90 H	−0.077 ± 0.019	(−0.076)	0.016 ± 0.043	(0.024)	−0.079 ± 0.039	(−0.106)
A	30 L	−0.030 ± 0.020	(−0.036)	−0.120 ± 0.035	(−0.149)	−0.009 ± 0.042	(0.002)
	30 M	0.003 ± 0.045	(−0.179)	−0.045 ± 0.050	(−0.015)	−0.066 ± 0.022	(−0.034)
	30 H	0.074 ± 0.015	(0.075)	−0.070 ± 0.037	(−0.118)	**−0.137 ± 0.034**	**(−0.102)**
	90 H	0.049 ± 0.041	(0.009)	0.035 ± 0.026	(0.058)	0.067 ± 0.090	(0.194)
AM	30 L	−0.025 ± 0.030	(−0.012)	−0.091 ± 0.039	(−0.050)	−0.045 ± 0.027	(−0.014)
	30 M	0.001 ± 0.041	(0.025)	−0.041 ± 0.060	(−0.021)	**−0.124 ± 0.024**	**(−0.116)**
	30 H	0.063 ± 0.040	(0.063)	−0.051 ± 0.049	(−0.098)	**−0.192 ± 0.038**	**(−0.123)**
	90 H	0.045 ± 0.060	(0.014)	0.031 ± 0.044	(0.007)	−0.011 ± 0.065	(0.004)
PM	30 L	−0.080 ± 0.019	(−0.103)	**−0.211 ± 0.032**	**(−0.174)**	**−0.182 ± 0.039**	**(−0.144)**
	30 M	−0.094 ± 0.031	(−0.067)	−0.206 ± 0.058	(−0.201)	**−0.257 ± 0.043**	**(−0.194)**
	30 H	−0.040 ± 0.041	(−0.002)	−0.230 ± 0.068	(−0.239)	**−0.300 ± 0.059**	**(−0.354)**
	90 H	−0.074 ± 0.030	(−0.057)	−0.108 ± 0.062	(−0.195)	−0.068 ± 0.065	(−0.038)
LM	30 L	**−0.098 ± 0.019**	**(−0.093)**	−0.128 ± 0.045	(−0.062)	−0.004 ± 0.027	(0.009)
	30 M	−0.094 ± 0.023	(−0.113)	−0.075 ± 0.055	(−0.002)	−0.110 ± 0.052	(−0.078)
	30 H	−0.073 ± 0.042	(−0.139)	−0.063 ± 0.047	(0.009)	**−0.139 ± 0.043**	**(−0.144)**
	90 H	−0.079 ± 0.025	(−0.106)	0.041 ± 0.055	(0–.078)	−0.011 ± 0.028	(−0.028)
RL	30 L	**−0.076 ± 0.013**	**(−0.062)**	**−0.138 ± 0.038**	**(−0.077)**	−0.070 ± 0.036	(−0.089)
	30 M	−0.064 ± 0.030	(−0.096)	−0.067 ± 0.062	(−0.001)	**−0.139 ± 0.027**	**(−0.188)**
	30 H	0.003 ± 0.026	(−0.036)	−0.082 ± 0.050	(−0.070)	**−0.216 ± 0.034**	**(−0.213)**
	90 H	−0.056 ± 0.027	(−0.086)	0.026 ± 0.047	(−0.001)	−0.031 ± 0.058	(−0.016)
RSC	30 L	−0.024 ± 0.012	(−0.051)	−0.063 ± 0.021	(−0.054)	−0.013 ± 0.009	(−0.022)
	30 M	−0.032 ± 0.015	(−0.034)	−0.024 ± 0.037	(−0.021)	−0.014 ± 0.015	(−0.071)
	30 H	0.023 ± 0.010	(0.036)	−0.067 ± 0.029	(−0.066)	−0.061 ± 0.023	(−0.046)
	90 H	−0.011 ± 0.019	(0.010)	0.026 ± 0.013	(0.035)	0.068 ± 0.017	(0.080)

*Values represent the cortical Amplitude response to the visual stimulation (n = 5) (30 L: 30°, 50%; 30 M: 30°, 75%; 30 H: 30°, 100%; 90 H: 90°, 100%) cortical response [means ± sem (median)], significant change, p ≤ 0.05 are represented in bold, t-test compared to ΔPre-Post cortical response (cV1, contralateral primary visual cortex; iV1, ipsilateral primary visual cortex; PM, posterior-median cortex; LM, latero-median cortex; A, anterior cortex; AL, anterio-lateral cortex; AM, anterio-median cortex; RL, rostro-lateral cortex; RS, retrosplenial cortex). The values corresponding to the conditioned stimulus (30 H) are underlined in gray*.

**Table 4:** Modification in the *Size* (ΔPost-Pre cortical response) in response of the visual stimulation.

**Area**	**Stim**.	**Treatment**					
		**Sham**		**CS**		**CS/DPZ**	
cV1	30 L	−0.004 ± 0.145	(−0.124)	−0.169 ± 0.190	(−0.301)	−0.048 ± 0.014	(−0.054)
	30 M	0.024 ± 0.166	(−0.128)	−0.009 ± 0.192	(−0.018)	−0.083 ± 0.030	(−0.046)
	30 H	0.022 ± 0.173	(−0.146)	−0.008 ± 0.198	(0.073)	**−0.088 ± 0.017**	**(−0.101)**
	90 H	−0.005 ± 0.162	(−0.157)	0.019 ± 0.183	(0.082)	0.006 ± 0.019	(−0.007)
iV1	30 L	−0.041 ± 0.054	(0.026)	−0.051 ± 0.069	(−0.155)	−0.009 ± 0.037	(−0.008)
	30 M	−0.100 ± 0.032	(−0.060)	0.077 ± 0.030	(0.091)	−0.013 ± 0.038	(−0.014)
	30 H	−0.090 ± 0.090	(−0.072)	0.062 ± 0.051	(0.067)	0.063 ± 0.024	(0.070)
	90 H	−0.086 ± 0.050	(−0.095)	0.076 ± 0.064	(0.035)	0.127 ± 0.041	(0.154)
AL	30 L	−0.217 ± 0.134	(−0.336)	−0.074 ± 0.171	(0.041)	−0.164 ± 0.071	(−0.068)
	30 M	−0.093 ± 0.129	(−0.143)	0.027 ± 0.165	(0.300)	**−0.228 ± 0.042**	**(−0.212)**
	30 H	−0.164 ± 0.156	(−0.344)	−0.029 ± 0.224	(−0.059)	−0.276 ± 0.065	(−0.328)
	90 H	−0.178 ± 0.147	(−0.414)	0.164 ± 0.200	(0.412)	−0.065 ± 0.139	(−0.213)
A	30 L	0.245 ± 0.095	(0.124)	0.131 ± 0.080	(0.108)	0.073 ± 0.152	(−0.008)
	30 M	0.195 ± 0.089	(0.058)	0.255 ± 0.081	(0.172)	−0.182 ± 0.073	(−0.164)
	30 H	0.214 ± 0.076	(0.171)	0.100 ± 0.138	(−0.021)	−0.045 ± 0.131	(−0.138)
	90 H	0.262 ± 0.113	(0.125)	0.263 ± 0.129	(0.101)	0.018 ± 0.154	(0.010)
AM	30 L	0.197 ± 0.114	(0.123)	0.178 ± 0.096	(0.223)	0.045 ± 0.130	(0.129)
	30 M	0.170 ± 0.134	(0.160)	0.217 ± 0.104	(0.235)	**−0.208 ± 0.031**	**(−0.191)**
	30 H	0.199 ± 0.116	(0.129)	0.127 ± 0.151	(−0.184)	−0.210 ± 0.101	(−0.246)
	90 H	0.231 ± 0.140	(0.080)	0.295 ± 0.131	(0.256)	−0.051 ± 0.125	(0.024)
PM	30 L	0.139 ± 0.133	(0.000)	−0.170 ± 0.180	(−0.316)	**−0.273 ± 0.033**	**(−0.239)**
	30 M	0.118 ± 0.149	(−0.014)	−0.034 ± 0.175	(−0.047)	**−0.268 ± 0.045**	**(−0.240)**
	30 H	0.143 ± 0.147	(0.000)	−0.088 ± 0.191	(−0.041)	**−0.260 ± 0.050**	**(−0.265)**
	90 H	0.150 ± 0.142	(−0.054)	0.048 ± 0.211	(0.099)	−0.076 ± 0.047	(−0.067)
LM	30 L	−0.059 ± 0.125	(−0.230)	−0.118 ± 0.181	(−0.230)	−0.044 ± 0.055	(−0.119)
	30 M	−0.058 ± 0.159	(−0.351)	0.075 ± 0.195	(0.049)	−0.124 ± 0.068	(−0.068)
	30 H	−0.141 ± 0.183	(−0.495)	0.087 ± 0.207	(0.053)	−0.099 ± 0.044	(−0.048)
	90 H	−0.142 ± 0.161	(−0.479)	0.175 ± 0.225	(0.117)	0.099 ± 0.066	(0.039)
RL	30 L	0.022 ± 0.132	(−0.127)	0.027 ± 0.161	(0.120)	−0.178 ± 0.095	(−0.257)
	30 M	0.095 ± 0.125	(−0.069)	0.090 ± 0.152	(0.234)	**−0.242 ± 0.057**	**(−0.304)**
	30 H	0.069 ± 0.140	(−0.044)	−0.077 ± 0.176	(−0.258)	−0.381 ± 0.104	(−0.598)
	90 H	0.054 ± 0.145	(−0.127)	0.177 ± 0.182	(0.143)	−0.070 ± 0.149	(0.017)
RSC	30 L	−0.038 ± 0.052	(−0.074)	−0.012 ± 0.082	(0.057)	−0.022 ± 0.054	(−0.065)
	30 M	−0.029 ± 0.055	(−0.119)	0.098 ± 0.052	(0.159)	−0.031 ± 0.043	(−0.009)
	30 H	0.068 ± 0.051	(0.051)	−0.030 ± 0.085	(−0.168)	−0.073 ± 0.039	(−0.062)
	90 H	−0.059 ± 0.090	(−0.227)	0.101 ± 0.094	(0.149)	0.134 ± 0.014	(0.132)

*Values represent the cortical Size response to the visual stimulation (n = 5) (30 L: 30°, 50%; 30 M: 30°, 75%; 30 H: 30°, 100%; 90 H: 90°, 100%) cortical response [means ± sem (median)], significant change, p ≤ 0.05 are represented in bold, t-test compared to ΔPre-Post cortical response (cV1, contralateral primary visual cortex; iV1, ipsilateral primary visual cortex; PM, posterior-median cortex; LM, latero-median cortex; A, anterior cortex; AL, anterio-lateral cortex; AM, anterio-median cortex; RL, rostro-lateral cortex; RS, retrosplenial cortex). The values corresponding to the conditioned stimulus (30 H) are underlined in gray*.

**Table 5:** Modification in the *Latency* (ΔPost-Pre cortical response) in response of the visual stimulation.

**Area**	**Stim**.	**Treatment**					
		**Sham**		**CS**		**CS/DPZ**	
cV1	30 L	1.800 ± 0.611	(2.000)	0.000 ± 0.699	(1.000)	0.400 ± 0.806	(0.000)
	30 M	1.400 ± 0.686	(1.000)	0.000 ± 0.558	(0.000)	−0.200 ± 1.020	(−2.000)
	30 H	1.000 ± 0.632	(1.000)	−0.600 ± 0.581	(−2.000)	0.600 ± 0.452	(0.000)
	90 H	2.000 ± 0.558	(2.000)	1.000 ± 1.054	(1.000)	−1.200 ± 0.998	(−1.000)
iV1	30 L	−1.600 ± 1.024	(−3.000)	1.800 ± 1.638	(5.000)	−2.800 ± 2.736	(−3.000)
	30 M	0.200 ± 0.998	(−1.000)	0.000 ± 0.760	(1.000)	−2.400 ± 0.618	(−2.000)
	30 H	0.200 ± 0.879	(0.000)	0.600 ± 0.777	(2.000)	0.800 ± 1.143	(0.000)
	90 H	−1.000 ± 0.869	(0.000)	0.200 ± 1.806	(−1.000)	−1.000 ± 0.789	(0.000)
AL	30 L	0.800 ± 1.718	(2.000)	2.000 ± 0.760	(2.000)	−0.600 ± 0.884	(−1.000)
	30 M	−0.200 ± 0.573	(0.000)	−0.600 ± 0.581	(0.000)	−0.600 ± 0.618	(−1.000)
	30 H	1.000 ± 0.422	(2.000)	−1.400 ± 1.258	(−2.000)	−1.400 ± 1.514	(−2.000)
	90 H	2.400 ± 0.542	(2.000)	0.800 ± 1.356	(2.000)	−2.600 ± 2.237	(−1.000)
A	30 L	0.800 ± 1.611	(−1.000)	1.400 ± 1.166	(2.000)	−1.800 ± 1.971	(−2.000)
	30 M	5.600 ± 1.694	(4.000)	1.000 ± 2.055	(−1.000)	0.200 ± 1.254	(−1.000)
	30 H	1.800 ± 0.827	(1.000)	3.200 ± 1.855	(2.000)	−4.000 ± 1.660	(−5.000)
	90 H	−2.000 ± 1.174	(−2.000)	1.800 ± 1.451	(2.000)	−4.200 ± 1.718	(−5.000)
AM	30 L	2.200 ± 0.533	(2.000)	−1.400 ± 1.046	(−1.000)	−0.400 ± 1.939	(−1.000)
	30 M	1.600 ± 0.581	(3.000)	4.000 ± 1.592	(3.000)	0.800 ± 1.236	(3.000)
	30 H	3.000 ± 1.414	(3.000)	1.600 ± 0.653	(0.000)	−4.600 ± 1.833	(−3.000)
	90 H	0.000 ± 1.193	(−2.000)	0.400 ± 1.833	(−1.000)	−2.000 ± 2.211	(1.000)
PM	30 L	2.200 ± 0.490	(3.000)	0.000 ± 0.596	(0.000)	−0.200 ± 0.442	(0.000)
	30 M	2.000 ± 0.843	(3.000)	−0.400 ± 0.400	(−1.000)	0.200 ± 0.929	(−1.000)
	30 H	0.200 ± 0.533	(−1.000)	0.000 ± 0.558	(1.000)	0.400 ± 0.653	(−1.000)
	90 H	1.000 ± 0.966	(1.000)	0.200 ± 1.482	(−2.000)	0.600 ± 1.002	(1.000)
LM	30 L	1.400 ± 1.408	(1.000)	−1.200 ± 1.181	(−3.000)	−2.200 ± 0.327	(−3.000)
	30 M	−0.800 ± 0.742	(−1.000)	−0.400 ± 0.618	(−1.000)	−1.000 ± 0.471	(−1.000)
	30 H	2.200 ± 0.646	(2.000)	−1.400 ± 0.542	(−1.000)	0.000 ± 0.760	(0.000)
	90 H	1.400 ± 0.618	(1.000)	2.000 ± 1.789	(1.000)	−2.800 ± 1.555	(−2.000)
RL	30 L	1.400 ± 1.586	(3.000)	−0.800 ± 1.597	(−4.000)	0.200 ± 1.541	(1.000)
	30 M	−1.600 ± 0.833	(−1.000)	−0.800 ± 0.389	(−1.000)	−1.800 ± 0.975	(−1.000)
	30 H	0.400 ± 0.400	(1.000)	−0.200 ± 0.929	(−1.000)	−2.000 ± 1.874	(−2.000)
	90 H	−1.000 ± 1.155	(−2.000)	3.000 ± 1.414	(2.000)	2.000 ± 0.869	(1.000)
RSC	30 L	0.000 ± 1.054	(1.000)	−0.600 ± 0.806	(−1.000)	−1.800 ± 1.467	(−1.000)
	30 M	−0.200 ± 1.020	(−1.000)	1.000 ± 2.055	(−2.000)	1.000 ± 1.317	(2.000)
	30 H	1.800 ± 0.573	(1.000)	2.200 ± 0.573	(2.000)	−1.000 ± 0.816	(−1.000)
	90 H	−1.200 ± 1.569	(−2.000)	−0.800 ± 0.975	(−2.000)	0.800 ± 1.181	(2.000)

*Values represent the cortical Latency response to the visual stimulation (n = 5) (30 L: 30°, 50%; 30 M: 30°, 75%; 30 H: 30°, 100%; 90 H: 90°, 100%) cortical response [means ± sem (median)], There were no significant differences between groups. (cV1, contralateral primary visual cortex; iV1, ipsilateral primary visual cortex; PM, posterior-median cortex; LM, latero-median cortex; A, anterior cortex; AL, anterio-lateral cortex; AM, anterio-median cortex; RL, rostro-lateral cortex; RS, retrosplenial cortex). The values corresponding to the conditioned stimulus (30 H) are underlined in gray*.

**Table 6:** Modification in the *SNR* (ΔPost-Pre cortical response) in response of the visual stimulation.

**Area**	**Stim**.	**Treatment**					
		**Sham**		**CS**		**CS/DPZ**	
cV1	30 L	**−5.504 ± 0.939**	**(−6.355)**	−1.907 ± 1.768	(−3.996)	**−3.306 ± 0.501**	**(−5.837)**
	30 M	−4.327 ± 1.539	(−4.141)	−3.725 ± 3.237	(−2.985)	**−4.766 ± 0.662**	**(−11.005)**
	30 H	−5.766 ± 1.486	(−8.209)	2.983 ± 2.863	(−0.047)	−9.187 ± 1.766	(−2.867)
	90 H	−2.594 ± 0.959	(−2.473)	7.242 ± 3.414	(3.423)	−1.487 ± 1.629	(0.603)
iV1	30 L	0.058 ± 0.442	(0.440)	−0.854 ± 0.508	(−0.211)	0.367 ± 0.178	(0.085)
	30 M	−1.101 ± 0.571	(−0.779)	0.340 ± 0.628	(1.567)	0.295 ± 0.430	(−1.238)
	30 H	0.499 ± 0.709	(1.279)	2.775 ± 0.818	(2.652)	−0.609 ± 0.485	(0.623)
	90 H	−1.934 ± 0.938	(−2.564)	2.819 ± 0.750	(2.850)	1.653 ± 0.981	(−2.278)
AL	30 L	**−4.161 ± 0.917**	**(−3.268)**	−3.528 ± 1.243	(−3.304)	−2.162 ± 1.031	(−2.411)
	30 M	−3.300 ± 0.930	(−3.170)	−0.658 ± 1.554	(−1.325)	**−2.020 ± 0.321**	**(−4.938)**
	30 H	−2.525 ± 1.475	(−2.129)	0.056 ± 1.580	(−0.851)	**−5.234 ± 0.403**	**(−0.635)**
	90 H	−3.407 ± 1.386	(−4.288)	2.025 ± 0.734	(1.851)	−0.655 ± 1.501	(0.031)
A	30 L	−0.144 ± 1.189	(−0.480)	**−2.874 ± 0.800**	**(−1.170**)	0.773 ± 1.161	(−0.308)
	30 M	−0.512 ± 1.363	(−0.520)	−1.088 ± 0.935	(−0.018)	−0.408 ± 0.627	(−1.397)
	30 H	2.965 ± 0.779	(2.308)	−0.354 ± 0.667	(−0.769)	**−1.519 ± 0.226**	**(0.332)**
	90 H	1.111 ± 1.156	(0.113)	4.870 ± 1.985	(2.017)	1.008 ± 1.144	(−0.019)
AM	30 L	0.185 ± 1.218	(−0.603)	−1.956 ± 1.227	(−0.496)	−0.877 ± 1.074	(−1.856)
	30 M	−0.361 ± 1.240	(−1.507)	−0.606 ± 1.568	(−0.028)	**−2.107 ± 0.452**	**(−2.772)**
	30 H	2.696 ± 1.585	(2.715)	0.194 ± 1.310	(−1.336)	**−3.147 ± 0.524**	**(0.173)**
	90 H	1.131 ± 1.599	(1.388)	4.603 ± 1.724	(3.499)	−0.831 ± 1.035	(−2.344)
PM	30 L	−1.261 ± 0.916	(−2.382)	−3.458 ± 1.226	(−5.494)	**−3.958 ± 0.818**	**(−3.910)**
	30 M	−0.477 ± 0.847	(0.156)	−3.034 ± 1.772	(−5.262)	**−4.272 ± 0.556**	**(**–**8.087)**
	30 H	0.054 ± 1.575	(2.053)	0.177 ± 1.799	(−0.835)	**−6.313 ± 1.139**	**(**–**2.341)**
	90 H	−0.384 ± 1.063	(−0.400)	6.196 ± 2.226	(4.214)	−0.942 ± 1.939	(−0.114)
LM	30 L	**−4.189 ± 0.472**	**(**–**4.626)**	−1.521 ± 1.612	(−1.382)	0.095 ± 0.730	(−1.288)
	30 M	−3.416 ± 1.276	(−4.333)	−0.116 ± 2.088	(0.060)	−1.540 ± 0.706	(−6.654)
	30 H	−4.055 ± 1.884	(−6.771)	2.858 ± 2.120	(0.285)	−5.189 ± 1.370	(−0.990)
	90 H	−3.593 ± 0.825	(−5.035)	7.007 ± 3.201	(0.956)	1.232 ± 1.013	(−2.942)
RL	30 L	−1.623 ± 1.146	(−0.233)	**−3.190 ± 0.893**	**(−2.021**)	−1.334 ± 1.462	(−1.824)
	30 M	−2.034 ± 1.059	(−2.743)	−1.453 ± 1.530	(−0.398)	**−1.982 ± 0.200**	**(−3.940)**
	30 H	0.586 ± 1.028	(0.708)	−1.045 ± 1.074	(−1.691)	**−4.368 ± 0.332**	**(1.019)**
	90 H	−1.250 ± 1.559	(−2.217)	3.876 ± 1.626	(2.352)	−0.132 ± 1.395	(0.334)
RSC	30 L	−0.883 ± 0.622	(0.254)	−2.924 ± 0.758	(−2.125)	0.274 ± 0.566	(−0.936)
	30 M	−1.326 ± 0.450	(−1.482)	−0.712 ± 0.956	(−1.337)	−0.043 ± 0.423	(−2.072)
	30 H	0.820 ± 0.429	(1.683)	−0.373 ± 0.871	(−1.383)	−1.210 ± 0.584	(0.513)
	90 H	−0.002 ± 0.914	(−0.129)	2.018 ± 0.884	(1.010)	0.285 ± 0.594	(−5.837)

*Values represent the cortical signal-noise-ratio (SNR) response to the visual stimulation (n = 5) (30 L: 30°, 50%; 30 M: 30°, 75%; 30 H: 30°, 100%; 90 H: 90°, 100%) cortical response [means ± sem (median)], significant change, p ≤ 0.05 are represented in bold, t-test compared to ΔPre-Post cortical response (cV1, contralateral primary visual cortex; iV1, ipsilateral primary visual cortex; PM, posterior-median cortex; LM, latero-median cortex; A, anterior cortex; AL, anterio-lateral cortex; AM, anterio-median cortex; RL, rostro-lateral cortex; RS, retrosplenial cortex). The values corresponding to the conditioned stimulus (30 H) are underlined in gray*.

For the CS group, the changes elicited by the conditioning were highly variable between mice. The cortical response to the conditioned stimulus (30 H) was in some cases reduced post-conditioning compared to the pre-values for every contrast (30 L, 30 M, and 30 H), but this change was not significant (Figure 4B). In contradistinction, activation in the ipsilateral V1 (iV1) was measured when presenting the highest contrast stimuli (30 and 90 H). This activation was located in the upper-lateral region of iV1, corresponding to the binocular region of this cortex (Figure 2). There was no observable modification of the CaS after the 1-week conditioning for the non-conditioned stimulus (90 H, Figures 4A–D). The monocular visual conditioning caused a significant decrease in the *Amplitude* on D8 for the lowest contrast of the conditioned orientation (30 L), only in the cV1, PM, and RL cortices (Table 3). The modification in the *Size* (Table 4) of the responses was highly variable between mice in AL (Figure 4B) and LM (Figure 4D), but none were significantly diminished. The *Latency* (Table 5) was not significantly modified in any areas for any stimulation. The *SNR* (Table 6) was significantly reduced in response to the lowest stimulation contrast (30 L) in A and RL.

For the CS/DPZ group, the variability of the results was much lower. The *Amplitude* in response to the conditioned stimuli was significantly decreased on D8 in cV1 (Figure 4A) and PM (Figure 4C) (30 L, 30 M, and 30 H), AM and RL (30 M and 30 H), and AL (Figure 4B) and LM (Figure 4D) (30 H) (Table 3). The non-conditioned stimulus (90 H) was not affected in any visual areas (Figure 4). The comparison of the response amplitude of both orientations (30 vs. 90 H) at D8 showed a significant difference in iV1, AM, PM, RL, and RSC following the DPZ treatment, whereas this difference was not observable on D0. Additionally, the Friedman analysis showed that the *Amplitude* response to the different contrasts (30 L, 30 M, and 30 H) was not significant after the treatment in cV1, AL, PM, and LM (Figure 4), even though it was before the conditioning. The *Size* was significantly reduced in response to the CS/DPZ group in cV1 (Figure 4A) (30 H), AM and RL (30 M), and in the PM cortex (30 L, 30 M, and 30 H, Figure 4C). A significant difference in the *size* between both orientations (30 vs. 90 H) was observable on D8, only in PM. Finally, the cholinergic potentiation of the conditioning had a significant influence on the *SNR* (Table 6) in multiple visual cortices and the PM cortex (30 L, 30 M, and 30 H, Figure 4C), as well as in cV1 (30 L and 30 M), AL (Figure 4B), AM and RL (30 M and 30 H), and A (30 H). The *Latency* and the *Persistence* were not affected following the cholinergic potentiation. Finally, no significant modification of any CaS parameters was observed in response to the 90 H stimulation (Figure 4, Tables 3–6).

When comparing of the post-pre variation, the *Amplitude* was significantly modified by treatments in PM for all contrasts of the conditioned orientation (30 L, 30 M, and 30 H), but not for the non-conditioned stimulus (90 H). The multiple comparisons showed that this modification occurred between the non-conditioned group and the DPZ conditioning group, while no significant difference was observable between both conditioned groups. This modification was also observable in AL (30 M and 30 H) and RL (30 H). Despite the Kruskal-Wallis test not showing any significant effect of the treatments in cV1 and AM, multiple comparisons showed a clear significant effect between non-conditioned and DPZ groups (30 H). The only significant differences between CS and CS/DPZ groups were observed in RL and AL for the conditioned stimulus (30 H). For the *Size*, our results showed that there was a significant modification in PM for two contrasts of the conditioned stimulus (30 M and 30 H), both significant diminutions occurring between the non-conditioned and the CS/DPZ group. For the *Latency*, only the response in AM was significantly changed for the conditioned stimulus (30 H), expressed by a significant diminution of *Latency* between the non-conditioned and the CS/DPZ group. Lastly, for the *SNR*, we observed a significant change for the conditioned stimulus (30 H) in cV1 and LM; this significant diminution of the *SNR* was shown between both conditioned groups (CS vs. DPZ). Interestingly, while the variation was low for the sham and CS/DPZ groups, the conditioning alone caused high interindividual variability in the treatment effect.

### Activation Correlations

**Figure 5:**
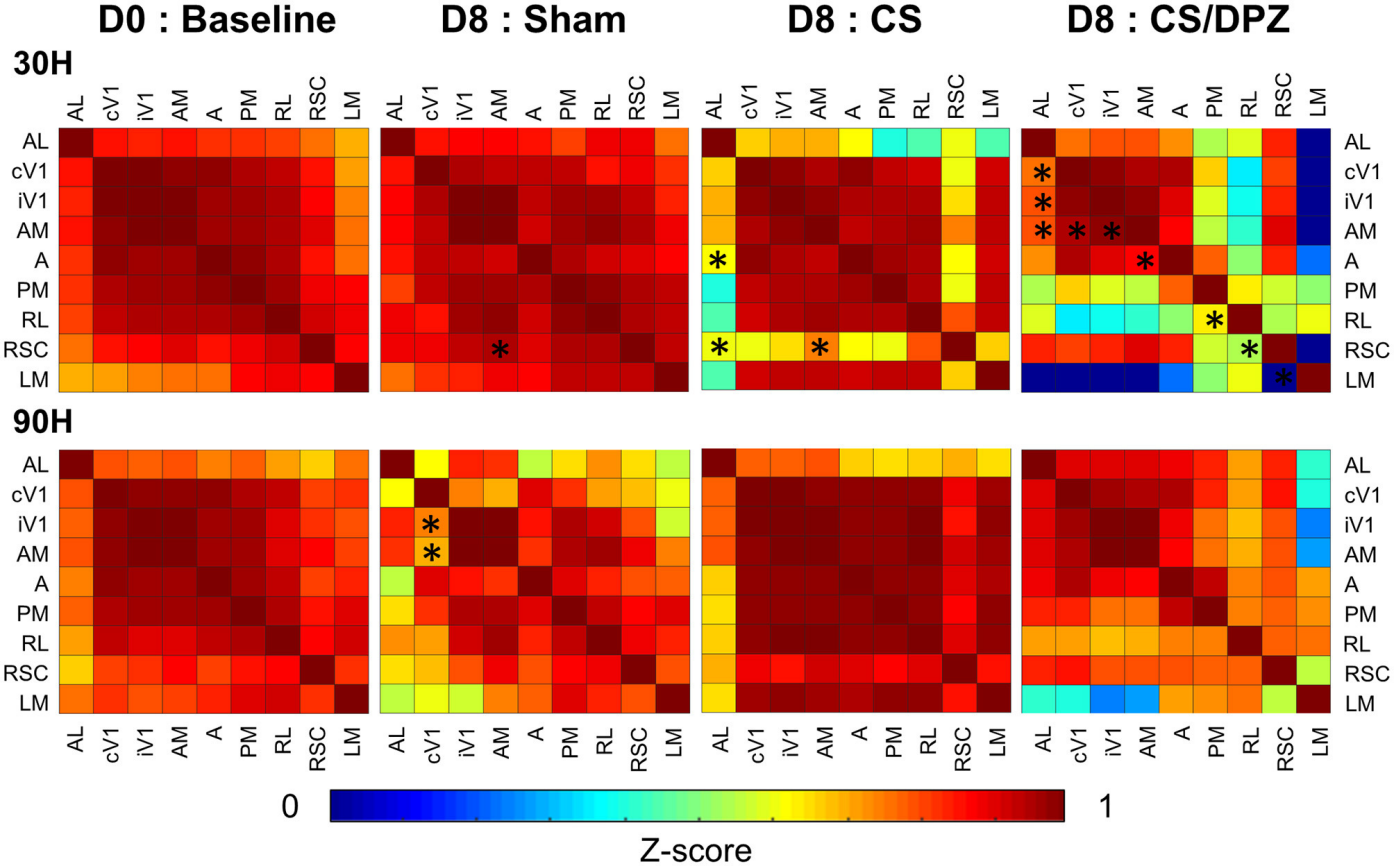
Activation correlation matrix of cortical visual areas. Conditioning weakens the activation correlation between V1 and RSC/AL/PM/A and between A/AM and PM in DPZ group while only between V1/AL and PM in CS. The correlation between AL and LM/PM is also weakened for the non-conditioned stimulus in CS but not in CS/DPZ group (*n* = 5 for each group, Fischer Z-transform and paired *T*-test, *p < 0.05, compared to the group baseline).

The co-activation of the cortical areas elicited by the conditioned (30°) or non-conditioned (90°) orientation presentation was evaluated using the Pearson's Correlation analysis (Figure 5) at D0 and D8. To highlight significant modifications in the activation correlation, results from both days were transformed using the Fisher Z-Transformation, allowing for *t*-test comparisons.

Before the conditioning, the activation correlation was similar for 30 H or 90 H. All selected areas of the visual system were strongly correlated with each other (*r* = 0.69–0.99), with the lowest correlation was expressed between the activation of AL and LM (*r* = 0.69) and the highest between cV1 and iV1 (*r* = 0.99). The correlation between cV1 and the ventral visual stream (A, AM, AL, PM, RL) was stronger (*r* = 0.84–0.98) than for the dorsal stream (LM; *r* = 0.70). After the conditioning, the highest effect for the conditioned stimulus (30 H) was seen in areas AL and RSC. In fact, a weaker correlation between both areas (and most of the cortical areas) was observed for this conditioned stimulus (30 H) in the CS group. However, the correlation diminution was significant only between AL and A/RSC, and between RSC and AM. No substantial alteration was observed in response to the non-conditioned stimulus (90 H). Interestingly, while using DPZ, this decorrelation between RSC and visual areas was not observable, except for in PM and RL. Despite the lack of significance, the correlation between LM and other areas was heavily diminished (*r* = −0.25 vs. 0.60). A similar but weaker decorrelation was also observed between AL, PM, RL, and most of the cortical visual areas. However, the activation correlation was generally diminished in this group for the non-conditioned stimulus (90 H), but those changes were discrete and not significant. Comparing the post-conditioning activation correlation of both conditioned groups (CS vs. CS/DPZ), there were changes in the correlation, but none were significant in response to each stimulus (30 and 90 H) (Figure 5). For the sham group, there were also rare isolated changes, i.e., the activation correlation for the 30 H stimulus was significantly decreased between RSC and AM, and between cV1 and both iV1 and AM for the non-conditioned stimulus (90 H).

### Resting State Correlations

**Figure 6:**
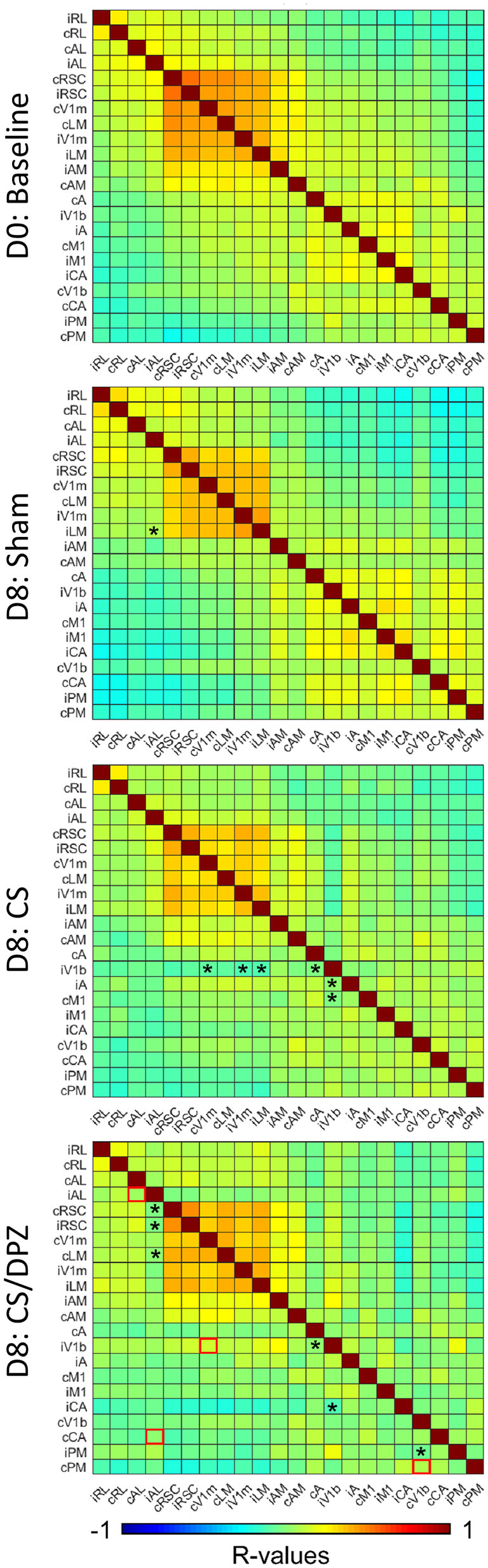
Resting state correlation. Conditioning with saline weakens the correlation of ipsilateral binocular V1 (iV1b) with cV1M, cA, cM1, and iV1M. Injection of DPZ during the conditioning diminish this effect on iV1b, while in DPZ group, only the correlation between iV1b and cA and cM1 are weakened (*n* = 5 for each group, Fischer Z-transform and paired *T*-test, *p < 0.05, compared to the baseline; unpaired *T*-test, red square=*p* < 0.05, comparing D8 CS to D8 CS/DPZ resting state correlation).

To evaluate the effect of conditioning on the resting state activity, which may reflect the long-term modification of the cortical network occurring in response to the conditioning, the change in the correlation between CaS was measured at rest. Our results showed that the monocular visual conditioning weakened the correlation between the binocular region of the ipsilateral V1 (iV1b) and cV1m, cA, iV1m, iLM, and iA. The cholinergic potentiation through DPZ injection during the conditioning partially restored the correlation between both hemispheres. We observed a diminution of the correlations only between iV1b and both cA and iAC in this group. While comparing the post-conditioning for both conditioned groups, a significant change in correlation between both hemispheres' AL, iV1b, and cV1m, and between cPM and cV1b was observed. For the non-conditioned group, there was no major modification in the resting state correlation over the experiment period (Figure 6).

### Gene Expression Modification

**Figure 7:**
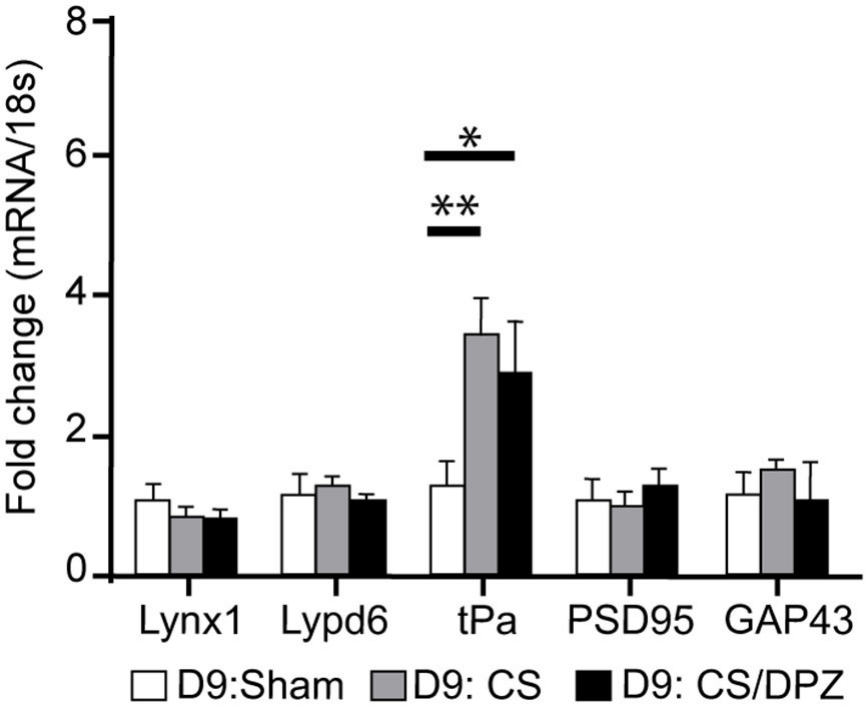
Gene expression modification through conditioning. Conditioning enhanced the expression of tPa in both conditioned groups but caused no modification in the expression of Lynx1, Lypd6, mAChR M2, PSD95, and GAP43 (*n* = 5 for each group, multiple *t*-test, *p = 0.004, **p = 0.0001, compared to Sham group).

The expression of plasticity markers was quantified by RT-qPCR after our treatment. Our results showed that the conditioning enhanced the expression of tPa in V1 for both conditioned groups (CS group = 3.45 ± 0.54, *p* = 0.0001; CS/DPZ group = 2.91 ± 0.72, *p* = 0.0005), but caused no modification in the expression of Lynx1, Lypd6, PSD95, and GAP43 compared to the non-conditioned group (Figure 7). While comparing both conditioned groups (CS vs. DPZ), no significant difference in tPa expression was observed (*p* = 0.6377), nor in any other gene's expression.

## Discussion

In this study, we examined the effects of a 7-day visual monocular conditioning on the mesoscopic map of the entire cortex, as well as cortical correlations with or without cholinergic potentiation via systemic DPZ administration (0.3 mg/kg). As the responses in other cortical areas were negligible, we centered our analysis on nine reactive cortical areas related to vision: A, AL, RL, AM, PM, LM, cV1, and iV1, as well as in the RSC. Our results showed that there was a neuronal activity decrease in the superficial layers after conditioning, enhanced by the DPZ treatment. The significant effects were located in the contralateral visual areas and in the RSC. The functional connectivity between visual areas also decreased following the conditioning potentiated by DPZ. However, those modifications were observed predominantly in the ventral visual pathway. Additionally, an upregulation of tPa, a proteolytic factor involved in plasticity, was observed in the conditioned V1 regardless of the cholinergic potentiation, suggesting the involvement of synaptic plasticity in the conditioning process.

This is the first report showing mesoscale CaI mapping in the cortex upon visual stimulation with full field drifting gratings, and after a visual conditioning. The CaS was increased by a visual stimulation and was sensitive to the contrast but not the orientation of the gratings. The CaS was strikingly restricted to the cortical areas involved in vision. These areas were highly correlated during visual stimulation with drifting patterns in naïve animals. However, the CaS was not increased by the visual stimulation of other areas, including the ipsilateral V1. The downstream neural transmission of V1 to the prefrontal cortex was thus not detectable in Thy1-GCaMP6s mice with these experimental conditions. In the resting state, the CaS was only slightly correlated between bilateral cortices, except in the case of the primary visual cortex. The main result shows a strong reduction in the CaS for the conditioned stimulus in most of the cortical areas after CS/DPZ treatment, although a slight tendency for a decreased signal was also seen with sham or CS conditions.

The decrease in cortical activity induced by CS/DPZ is surprising, as previous studies rather demonstrated an enhancement of visual-evoked activity in similar conditions (Cooke and Bear, 2010; Kang et al., 2014; Chamoun et al., 2016). First, this discrepancy might be explained by experimental considerations, i.e., the level of signal collection or arousal state of the animal. The mCaI technique using GP4.3 Thy1-GCaMP6s mice is an amalgamation of the CaS from excitatory cell bodies, axons, and dendrites located in the superficial layers covering large areas of the cortex. On the other hand, previous electrophysiology recordings have been restricted to one site of layer 4 in V1 and result from the firing of both excitatory and inhibitory neurons. Moreover, recordings in the present study were performed on awake animals, as opposed to animals under anesthesia as seen in previous studies, which may change the neuronal activity, connectivity, or responsiveness to cholinergic input (Galuske et al., 2019). Specifically, awareness and the behavioral states of the animal such as arousal, attention, and locomotion (Niell and Stryker, 2010; Pakan et al., 2016) may influence the duration, dynamics of the evoked response, and cortico-cortical interactions (Sellers et al., 2015). It is possible, although we did not measure it, that repetitive administration of DPZ might slightly change the brain states of the animal although not detected in sleep duration for rats at this dose (Ishida and Kamei, 2009). Finally, mCaI measures the global rather than single-cell response. Due to the salt-and-pepper organization of the neurons in the rodent V1, it is possible that the CaS was augmented in conditioned and tuned neurons, but this signal could have been masked by the suppression of activity in the more numerous non-conditioned and un-tuned neurons by ACh, which suppresses irrelevant neuronal activation (Castro-Alamancos and Oldford, 2002). The decrease of the fluorescent signal in superficial layers, rather than an enhancement in layer 4 neuronal activity, might also be explained by the functional organization of the visual cortex, particularly the layer-dependent neural activity. For example, a layer-specific response to visual stimulation and to cholinergic activation has been previously demonstrated (Obermayer et al., 2017; Yildirim et al., 2019). The excitatory effect of sensory input is stronger in layer 4 (Verdier and Dykes, 2001), which is explained by the endings of the thalamocortical fibers and by fewer GABAergic cells in this layer compared to layers 2/3 (Gonchar et al., 2007). The activation of the layers 2/3 depends on layer 4 feedforward input, layer 5 recurrent circuits, as well as layer 1 feedback from other cortical layers; this connectivity is orchestrated by the inhibition from GABAergic cells (Makino and Komiyama, 2015; D'souza et al., 2016). It is possible that, in our study, both conditioning groups (CS and DPZ-CS) showed inhibition of layer 2/3 pyramidal cells due to either this GABAergic drive or a top-down modulation. This is in line with the calcium activity diminution in layers 2/3 after passive visual conditioning that has been previously observed (Makino and Komiyama, 2015; Henschke et al., 2020). It is also well-documented that cholinergic influence differs from one layer to another according to the receptors involved (Disney et al., 2012; Pfeffer et al., 2013; Obermayer et al., 2017), causing a differential effect of ACh (Oldford and Castro-Alamancos, 2003; Giocomo and Hasselmo, 2007; Soma et al., 2013b; Shimegi et al., 2016; Minces et al., 2017). In addition, it is possible that conditioning partially reduced cell firing, or a reduction of the CaS results from afferent axons, including projecting fibers in layer 1 (although the contribution of axons in the mesoscale CaS recorded is probably minor).

Our findings thus agree with previous studies showing that the conditioning might increase activation of GABAergic neurons in sensory cortices (Gierdalski et al., 2001; Jiao et al., 2006, 2011), leading to an upregulation of the inhibitory drive (Tokarski et al., 2007; Saar et al., 2012; Mckay et al., 2013). This inhibitory drive has been demonstrated as essential for the induction of condition-dependent synaptic plasticity and its maintenance (Posluszny et al., 2015). It is therefore possible that conditioning reduces the number of activated excitatory neurons in layers 2/3 or their level of excitation, which would be exacerbated by ACh. Accordingly, ACh increases inhibitory drive and suppresses lateral spreading (Kimura and Baughman, 1997; Zinke et al., 2006; Obermayer et al., 2018). Furthermore, the spread of a CaS response to the visual stimulation (reduced size of activated area and restriction of the correlation to primary visual areas) was reduced only by CS/DPZ treatment, confirming previous results with ACh administration (Kimura et al., 1999; Silver et al., 2008). Our previous studies have also shown a strong dependency of cholinergic potentiation on M2-type muscarinic and nicotinic receptors (Kang et al., 2015) associated with GABAergic neurons (Disney and Aoki, 2008; Groleau et al., 2015). However, cholinergic fibers modulate various inhibitory circuits, i.e., feed-forward inhibition, lateral inhibition and disinhibition (Obermayer et al., 2018), so we cannot directly infer the effect on GABAergic circuits induced by enhanced cholinergic transmission from our experiment. On the other hand, a decreased response following conditioning might reflect an experience-dependent adaptation of neurons, in which the reduction of activity corresponds to an increase in neuronal efficiency, as the cortical response to visual stimulation is not affected in the upstream secondary cortical areas.

The CS/DPZ reduced the *amplitude* response to the conditioned stimulus in V1, AM, LM, AL, and RSC. The correlation of cortical areas that respond to the pattern stimulation was also affected by our treatment, but only in V1 and in the ventral pathway, while the dorsal path (represented by LM) remained unaffected. This is likely due to the visual stimulus used, i.e., drifting gratings, which are processed by the ventral pathway (Marshel et al., 2011; Smith et al., 2017). The greatest effects occur in V1 and PM, which is unsurprising considering the fact that neuron selectivity in V1 is essential to orientation and contrast changes (Glickfeld et al., 2013b) and because PM is one of the most innervated visual areas, along with LM and AL (Wang et al., 2012). The low temporal frequency of our stimuli (1 Hz) might explain why PM, which responds to low temporal but high spatial frequencies, expresses more modifications in its response post-conditioning than AL, which has preferential affinity to high temporal and low spatial frequencies. These results might also suggest that the temporal frequency of the stimulation has a greater effect on the mouse's neuronal tuning than the spatial frequency. In fact, our stimulation (S.F.: 0.03 cpd, T.F.: 1 Hz, sinusoidal grating) is closer to the preferred spatial frequency of AL (S.F.: 0.045 cpd) and the preferred temporal frequency of PM (T.F.: 1.2 Hz) (Andermann et al., 2011).

Aside from PM, the subsequent extrastriate visual area responses were not significantly affected, while V1 responses were reduced, suggesting an improved efficiency in V1 feedforward neurons projecting to those areas. The effect of the CS/DPZ did not seem to be related to the release of plasticity brakes Lynx1 and LypD6, which affects nicotinic transmission, since the expression of these molecules was not modified. The increased expression of tPa during CS, combined with a weakening of the conditioned-stimulus response, suggests the involvement of LTD and/or LTP mechanisms. In fact, this plasticity marker is well-known to be essential in experience-dependent plasticity (Mataga et al., 2004). Additionally, its expression is upregulated during long-term potentiation (Qian et al., 1993) and long-term depression (Calabresi et al., 2000). In contradistinction, the expression of GAP43, which has an influence on AMPA receptor endocytosis and LTD (Han et al., 2013), was not modified by any treatments. It is therefore possible that LTP was involved in the mechanism of conditioning, improving the efficiency of neurons in superficial layers. Despite our hypothesis, the effect of cholinergic potentiation does not seem to be related to the release of the plasticity brakes Lynx1 and LypD6, reducing nicotinic transmission, as the expression of these molecules were not modified. Knowing that the cholinergic system plays a key role in visual attentional processes (Herrero et al., 2008), the administration of DPZ might have contributed to an improved beneficial effect on visual transmission. Consequently, DPZ reduced the increased inter-individual variability in the CS groups, suggesting an attentional effect of increased levels of ACh.

DPZ also abolished the CS-induced decorrelation between interhemispheric binocular and monocular zones of V1 during resting state, suggesting an effect of ACh on binocular interaction. These results are also concomitant with a recent human study showing that DPZ administration reduces the ocular dominance shift normally observed after a monocular deprivation (Sheynin et al., 2019) and reduce interocular suppression (Sheynin et al., 2020). Knowing that the binocular response is influenced by multiple factors such as the thalamocortical input from both eyes, the GABAergic modulation, and the corticocortical projections, it might be further explained by the influence of ACh on each of these factors (Disney et al., 2012; Groleau et al., 2015; Vaucher et al., 2019). This result may reflect the modification in perceptual strength in the conditioned eye over the other in the binocular region as observed in a monocular deprivation experiment (Scholl et al., 2017).

In regard to present and previous results, we suggest that the global decrease observed in cortical calcium responses in the superficial layers of V1 and PM might be due to the attenuation of pyramidal neuron activation in layers 2/3, even if layer 4 is activated, and thus reduces conscious perception of the conditioned stimulus. Accordingly, a similar repetitive passive visual stimulation causes a reduction of the calcium signals to the passive stimulation, whereas a rewarded presentation leads to an increased calcium response (Makino and Komiyama, 2015; Henschke et al., 2020). This may result from a suppression of the neurons' response to the passive conditioned stimulus in layers 2/3 from direct activation of the GABAergic neurons by feedforward inputs, or through top-down feedback activation of layer 1 inhibitory interneurons (Makino and Komiyama, 2015). It is also possible that the decreased activity resulted from reduced attention or motivation in the mice. It is tempting to speculate that this reduction of neuronal response in superficial layers probably reflects the habituation of the neurons to irrelevant stimuli, and prevents the upstream processing of this stimulus to high order cortices. Accordingly, we do not report any change in the high-level cortical areas. In that case, this suppression would diminish the perception of this signal, in line with previous studies suggesting the attenuation of the conscious perception of redundant signals that are irrelevant for survival (Briggs et al., 2013; Galuske et al., 2019). It is, however, contradictory to the effect of ACh in previous studies. First, ACh usually mediates visual attentional processes (Herrero et al., 2008; Li et al., 2018), therefore improving ACh cortical levels should alleviate the suppression effect of passive conditioning. ACh is usually considered as responsible for shifting the dynamics of the cortical circuits to a signal significant mode and enhancing cue detection (Sarter and Lustig, 2020). Second, the coupling of passive visual stimulation to electrical stimulation of cholinergic neurons or DPZ administration has been shown to selectively improve the detection of the conditioned grating after the training. This is in favor of an improved perception. In this regard, the effect of ACh on neuronal plasticity is highly relevant. First, the increase of tPA in this study suggests an LTP process, which is in line with previous studies showing the triggering of long-lasting events by ACh in V1 (Kang and Vaucher, 2009). Altogether, our results and these studies could argue for a processing of this passive stimulus for an automatic mode that does not require attentional demand and neuronal resources, but rather relies on improve neuronal efficiency. This would be in line with Furey and Ricciardi results proposing improved circuitry dynamics by DPZ in humans (Furey et al., 2000; Ricciardi et al., 2013).

## Data Availability Statement

The datasets generated for this study are available on request to the corresponding author.

## Ethics Statement

The animal study was reviewed and approved by Comité de déontologie de l'expérimentation sur les animaux (CDEA) de l'université de Montréal.

## Author Contributions

GL performed the experiments, wrote the MATLAB script supervised by Labeo Technologies, analyzed the data, and wrote the first draft of the paper. RO contributed to RT-qPCR experiments. GL and EV designed the experiments, interpreted the results, and finalized writing. All authors contributed to the article and approved the submitted version.

## Conflict of Interest

The authors declare that the research was conducted in the absence of any commercial or financial relationships that could be construed as a potential conflict of interest.
